# Characterization and Investigation of Novel Benzodioxol Derivatives as Antidiabetic Agents: An In Vitro and In Vivo Study in an Animal Model

**DOI:** 10.3390/biom13101486

**Published:** 2023-10-06

**Authors:** Mohammed Hawash, Derar Al-Smadi, Anil Kumar, Barbara Olech, Paulina Maria Dominiak, Nidal Jaradat, Sarah Antari, Sarah Mohammed, Ala’a Nasasrh, Murad Abualhasan, Ahmed Musa, Shorooq Suboh, İrfan Çapan, Mohammad Qneibi, Hiba Natsheh

**Affiliations:** 1 Department of Pharmacy, Faculty of Medicine and Health Sciences, An-Najah National University, Nablus 00970, Palestine; nidaljaradat@najah.edu (N.J.); s11819807@stu.najah.edu (S.A.); s11819564@stu.najah.edu (S.M.); s11821626@stu.najah.edu (A.N.); m_abualhasan@najah.edu (M.A.); 2 Department of Chemistry, Faculty of Sciences, An-Najah National University, Nablus 00970, Palestine; derarsmadi@najah.edu; 3 Biological and Chemical Research Centre, Department of Chemistry, University of Warsaw, ul. Żwirki i Wigury 101, 02-089 Warsaw, Poland; a.kumar3@uw.edu.pl (A.K.); b.gruza@uw.edu.pl (B.O.); pdomin@chem.uw.edu.pl (P.M.D.); 4 Centre of New Technologies, University of Warsaw, ul. S. Banacha 2c, 02-097 Warsaw, Poland; 5 Department of Biomedical Sciences, Faculty of Medicine and Health Sciences, An-Najah National University, Nablus 00970, Palestine; ahmed.mosa@najah.edu (A.M.); s.sobuh@najah.edu (S.S.); mqneibi@najah.edu (M.Q.); 6 Department of Material and Material Processing Technologies, Technical Sciences Vocational College, Gazi University, 06560 Ankara, Turkey; irfancapan@gmail.com; 7 Basic and Engineering Sciences Central Laboratory Application and Research Center (GUTMAM), Gazi University, 06500 Ankara, Turkey

**Keywords:** benzodioxol, α-amylase, cytotoxicity, mice, microED, streptozotocin, in vivo

## Abstract

In this study, we synthesized benzodioxol carboxamide derivatives and investigated their antidiabetic potential. The synthesized compounds (**Ia-Ic** and **IIa-IId**) underwent characterization via HRMS, ^1^H-, ^13^CAPT-NMR, and MicroED. Their efficacy against α-amylase was assessed in vitro, while MTS assays were employed to gauge cytotoxicity across cancer and normal cell lines. Additionally, the antidiabetic impact of compound **IIc** was evaluated in vivo using a streptozotocin-induced diabetic mice model. Notably, **IIa** and **IIc** displayed potent α-amylase inhibition (IC_50_ values of 0.85 and 0.68 µM, respectively) while exhibiting a negligible effect on the Hek293t normal cell line (IC_50_ > 150 µM), suggesting their safety. Compound **IId** demonstrated significant activity against four cancer cell lines (26–65 µM). In vivo experiments revealed that five doses of **IIc** substantially reduced mice blood glucose levels from 252.2 mg/dL to 173.8 mg/dL in contrast to the control group. The compelling in vitro anticancer efficacy of **IIc** and its safety for normal cells underscores the need for further in vivo assessment of this promising compound. This research highlights the potential of benzodioxol derivatives as candidates for the future development of synthetic antidiabetic drugs.

## 1. Introduction

Diabetes is a widespread, chronic condition that impacts populations across the globe. In 2010, approximately 285 million adults were estimated to have diabetes. The global prevalence of diabetes is expected to continue increasing, being primarily driven by factors like aging populations and overall population growth [1]. Diabetes mellitus is a metabolic anomaly characterized by elevated blood sugar levels. The disease is demarcated into two principal classifications: type I and type II. Being predominantly prevalent among the young, type I diabetes constitutes 5% to 10% of all diabetes cases. The annual incidence rate in East Asia is approximately 1 new case per 100,000 individuals [2].

Considerable efforts have been made to explore novel synthetic antidiabetic agents. Iminosugar-related compounds, glucosidase inhibitors, have captured special attention due to their good safety and efficacy profiles [3,4,5,6]. Molecules based on aromatic heterocyclic scaffolds, such as derivatives of coumarin, imidazole, pyrimidine, thiazole, and stereopure, are examples of commonly investigated compounds [7,8]. 

Alpha-amylase catalyzes the intricate metabolic process of converting starch (Latin amylum) into more easily digestible sugars. This enzyme can be found in human and other mammals’ saliva, where it starts the chemical digestion process [9,10]. Alpha-amylase inhibitors can prevent the enzyme alpha-amylase from breaking down complex carbs into simpler sugars. These inhibitors have the potential to help manage blood sugar levels and are being studied for their potential health benefits [11,12]. 

One such method is insulin and insulin-like growth factor (IGFs) control. Alpha-amylase inhibitors can assist in regulating blood sugar levels, which can influence insulin and IGF signaling pathways. Insulin and IGFs have been linked to an increased risk of some malignancies due to their role in cancer cell development and proliferation. Alpha-amylase inhibitors may influence cancer growth and progression by influencing various pathways [13,14,15].

The fundamental 1,3-benzodioxol core structure discovered within safrole occurs naturally in plants like *Crocus sativus* L. (saffron) and Ocotea pretiosa Mez. This structure showcases captivating biological activities. Scientists have leveraged this benzodioxole nucleus in drug exploration to create innovative compounds encompassing various biological impacts. These include combating schistosomiasis, countering epilepsy, alleviating pain, battling tuberculosis, and demonstrating antimicrobial prowess. The presence of this core structure in natural sources and its versatility in creating biologically active compounds make it a promising target for further research and pharmaceutical development [16,17,18,19,20,21,22,23].

**Figure 1:**
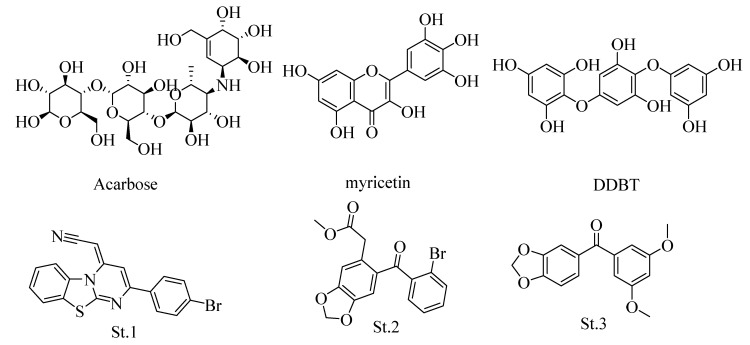
Structures of α-amylase inhibitors.

Numerous alpha-amylase inhibitors have been identified and grouped into various classes. These classes include carbohydrate-based α-amylase inhibitors like acarbose (Figure 1), with an IC_50_ value of 2.593 µM [24], and polyphenols like myricetin (Figure 1), with an IC_50_ value of 30 µM [25]. In 2012, a notable tannin known as phlorotannin 2-(4-(3,5-dihydroxyphenoxy)-3,5-dihydroxyphenoxy) benzene-1,3,5-triol (DDBT) emerged, revealing its remarkable prowess as an α-amylase inhibitor. Impressively, it demonstrated an IC_50_ value of 8.5 µM, solidifying its potent inhibitory capacity [26]. Moreover, among synthetic thiazolidine derivative compounds, **St.1** showed significant inhibitory activities against this enzyme with an IC_50_ value of 15.26 µM [27]. Our diligent research team had previously synthesized benzodioxole derivatives, subjecting them to α-amylase screening. Notably, **St.2** and **St.3** (as depicted in Figure 1) emerged as standout performers within this array of compounds. They showcased remarkable inhibitory potential, revealing IC_50_ values of 2.57 and 4.28 µg/mL, respectively, against this enzymatic target [28].

Based on the knowledge gained from these findings and recognizing the structural resemblance of polymethoxy groups found in polyphenols and benzodioxoles, researchers ventured to synthesize novel benzodioxole compounds and assess their interaction with this enzyme. This exploration aimed to identify more effective α-amylase inhibitor candidates, which could be utilized for various applications in managing conditions related to carbohydrate metabolism, such as diabetes and obesity.

## 2. Materials and Methods

### 2.1. Materials

The chemicals used in this study were obtained from well-regarded suppliers, such as Chemicals Company, Sigma-Aldrich, and Alfa Aesar. The substances were used in their original state, eliminating the need for additional purification, and included the following: 3,4-(Methylenedioxy)phenylacetic acid (Sigma Aldrich, Burlington, MA, USA; catalog #329673), 1,3-Benzodioxole-5-carboxylic acid (Sigma Aldrich, Burlington, MA, USA; catalog #P49805), 1-(3-Dimethylaminopropyl)-3-ethylcarbodiimide hydrochloride (EDC) (Alfa Aesar, Karlsruhe, Germany; catalog #A10807), 4-(Dimethylamino)pyridine (DMAP) (Sigma Aldrich, Glasgow, UK; catalog #39405-50G), 3,4,5-Trimethoxyaniline (Sigma Aldrich, Burlington, MA, USA; catalog #T68209-10G), silica gel (Sigma Aldrich, Beijing, China; catalog #S74874), 3,5-Dimethoxyaniline (Sigma Aldrich, Darmstadt, Germany; catalog #D130001), aniline (Sigma Aldrich, Darmstadt, Germany; catalog #132934), 2,4-Dimethoxyaniline (Sigma Aldrich, Glasgow, UK; catalog #D129801), 2,5-Dimethoxyaniline (Sigma Aldrich, Beijing, China; catalog #112984), 4-(2-Methoxyphenoxy)aniline (Sigma Aldrich, Burlington, MA, USA; catalog #H32565), 3-(Trifluoromethyl)aniline (Sigma Aldrich, Glasgow, UK; catalog #A15910), and streptozotocin (Sigma Aldrich, Burlington, MA, USA; catalog #S0130). DMEM media (Sigma Aldrich, Darmstadt, Germany; catalog #D0822), L-Glutamine solution (Sigma Aldrich, Glasgow, UK; catalog #D0822), and dinitrosalicylic acid (DNSA) (Sigma Aldrich, Burlington, MA, USA; catalog #128848) were also used.

### 2.2. Instrumentation

The synthesized compounds’ ^1^H and ^13^C NMR spectra were analyzed using a Bruker DPX-500 High-Performance Digital FT-NMR Spectrometer (Billerica, MA, USA) in solutions of DMSO-d_6_. The ^1^H-NMR spectra were observed at a 500 MHz frequency and the ^13^C-NMR spectra at 125 MHz. The resulting chemical shift values (δ) were gauged in parts per million (ppm), with coupling constants denoted in hertz (Hz). A water LCT Premier XE Mass Spectrometer (Waters Corporation, Milford, MA, USA) was utilized for the high-resolution mass spectra (HRMS) data, applying the electrospray ionization (ESI) technique in the positive polarity mode. This study was conducted at the Pharmacy Faculty of Gazi University in Ankara, Turkey.

#### 2.2.1. General Procedure for the Synthesis of Benzodioxol-Carboxamide (**Ia-IIe**)

**Scheme 1:**
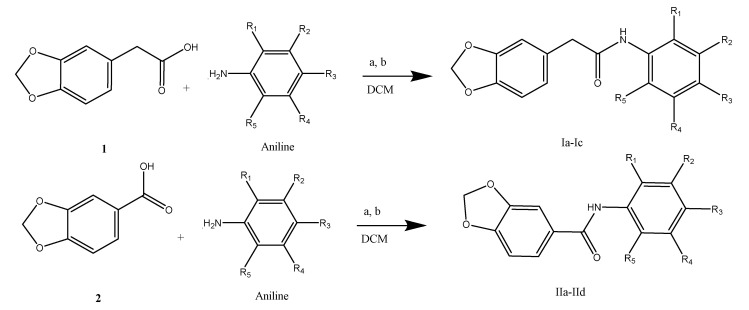
Upon stirring **1** or **2** starting materials along with the aniline derivatives within 20 mL of DCM, the subsequent steps were executed as follows: (a) the introduction of DMAP, and (b) the addition of EDC. Both steps were carried out under an argon gas environment. The resulting mixture was then stirred for a duration ranging between 48 and 72 h.

Following Scheme 1, a series of derivatives, **Ia-Ic** and **IIa-IIe**, were synthesized, including benzodioxole carboxamide compounds. Each acid (300 mg, 1.203 mmol) was dissolved in 15 mL of dichloromethane (DCM; 0.235 mmol) in a pristine round-bottom flask. Next, DMAP (4-Dimethylaminopyridine) (45 mg, 0.361 mmol) was added and stirred under argon to prevent oxidation. Following a short interval of 5–10 min, EDCI (1-ethyl-3-(3-dimethylaminopropyl)carbodiimide) (305.91 mg, 1.5639 mmol), a coupling reagent, was introduced. The reaction progressed under argon for 30 min. The fitting aniline derivative successfully merged into the reaction and was stirred for 48 h using a stirrer. TLC papers tracked the reaction; one was treated with ninhydrin for aniline detection, and the other was bromocresol-treated to affirm acid presence, ensuring product purity.

The reaction mixture was subsequently subjected to extraction using diluted HCl through a separatory funnel. The lower layer containing the aniline derivative was collected within a conical flask. Anhydrous sodium sulfate was introduced as a drying agent to eliminate residual moisture, and the resulting mixture was filtered using filter paper. Silica gel (3–4 spatulas) was incorporated into the filtrate, and then, taking into account the boiling point of DCM (39.6 °C), the solvent was evaporated utilizing a rotary vacuum evaporator. This process yielded a silica-loaded flask containing the product. For further purification, a silica gel column chromatography technique was employed. A solvent system of DCM/ethyl acetate (1:1) was utilized for this purpose. The tubes containing the product were collected and subsequently subjected to an evaporation step under vacuum [29].

#### 2.2.2. MicroED Sample Preparation, Data Collection, and Refinement Procedure

After synthesis, the crystalline powder of compounds **IIc** and **IId** was obtained directly without further crystallization processes. A small portion of the sample was first ground using a mortar. Grids for MicroED data collection were created by directly applying the powdered compounds to glow-discharged lacey carbon 200-mesh Cu grids. Data were collected using Thermo Fisher Scientific’s Galcios cryo-transmission electron microscope (TEM), operated at 200 kV and −192 °C. The TEM was outfitted with a field emission gun, a CETA-D detector from Thermo Fisher Scientific (Waltham, MA, USA), and an autoloader that could hold twelve grids and used EPU-D software (versions 1.15) for automated collection. The settings were adjusted to include a 50 μm condenser aperture, spot size 11, and gun lens 8. Diffraction datasets were gathered under parallel illumination using a very low dose. During the collection process, the crystal was consistently rotated from −60° to +60°, and the microscope was set to diffraction mode. Images were collected continuously in rolling shutter mode with hardware binning 2 and an exposure time of 0.5 s. These images were then converted to the SMV format, along with the essential metadata for processing in standard crystallographic programs.

Frames were indexed and integrated in XDS, and the intensities were converted to SHELX format using XDSCONV [30,31]. The structures were solved in SHELXT [32] and refined using Olex2 [33] with the application of kinematical diffraction theory. After standard IAM refinement in olex2.refine, the TAAM refinement was applied using the MATTS data bank through the DiSCaMB utility program (discambMATTS2tsc.exe version 2.006) [34,35] and the NoSpherA2 [36] module of Olex2. It was shown that TAAM refinement leads to better quality structural refinements than IAM [37]. Determining non-hydrogen atoms involved a meticulous process of successive difference Fourier syntheses, followed by refinement incorporating anisotropic thermal parameters. Calculated positions guided the positioning of hydrogen atoms, and their optimization was achieved through a riding model skillfully managed using suitable HFIX commands. To ensure accuracy, the X-H bond lengths were judiciously constrained to closely correspond with the mean values extrapolated from data obtained through neutron diffraction. This comprehensive approach underscores the precision and rigor applied in elucidating the atomic structure of the compounds under investigation.

#### 2.2.3. MicroED Method

Compound **IIc** was crystallized in a monoclinic system featuring a non-centrosymmetric space group, Cc, and the following lattice parameters: a = 7.1 Å, b = 6.8 Å, c = 24.3 Å, and β = 91.9°. Within the asymmetric unit, it contained a single molecule. Determining the absolute structure of this compound from 3D ED data through kinematical refinement was not feasible. Meanwhile, compound **IIe** crystallized within the centrosymmetric space group of the monoclinic crystal system, C2/c, accompanied by the lattice parameters of a = 28.3 Å, b = 4.9 Å, c = 20.4 Å, and β = 114.8°, and it also contained one molecule in its asymmetric unit. The R1 factors for compounds **IIc** and **IIe** were recorded at 10.60% and 14.19%, respectively, consistent with typical results for kinematical refinement on 3D ED data involving organic crystals.

Additional crystallographic details related to this study are accessible via CCDC entries 2288354 and 2288355. These data can be freely obtained at www.ccdc.cam.ac.uk/data_request/cif (accessed on 18 August 2023) or by submitting an email request to data_request@ccdc.cam.ac.uk. Alternatively, the Cambridge Crystallographic Data Centre can be contacted at 12 Union Road, Cambridge CB2 1EZ, UK, or via fax at +44 1223 336033.

**2-(benzo[d][1,3]dioxol-5-yl)-N-(4-chloro-2,5-dimethoxyphenyl)acetamide (Ia)**

For the compound 2-(benzo[d][1,3]dioxol-5-yl)-N-(4-chloro-2,5-dimethoxyphenyl)acetamide (**Ia**), the IR (FTIR/FTNIR-ATR) analysis shows an amide carbonyl (C=O) at 1675.38 cm^−1^. Its ^1^H NMR (500 MHz, DMSO) is δ 9.30 (s, 1H), 7.95 (s, 1H), 7.13 (s, 1H), 6.96–6.74 (m, 3H), 5.99 (s, 2H), 3.81 (s, 3H), 3.74 (s, 3H), 3.67 (s, 2H), and the ^13^C NMR (126 MHz, DMSO) is δ 170.18, 148.51, 147.60, 146.40, 143.90, 129.96, 127.59, 122.79, 115.37, 113.61, 110.17, 108.55, 106.97, 101.26, 57.11, 56.79, 42.92. HRMS (*m*/*z*): [M + H] + was calculated for C_17_H_17_ClNO_5_ as 350.0795, which was found to be 350.0796.

**2-(benzo[d][1,3]dioxol-5-yl)-N-phenylacetamide (Ib)**

The compound 2-(benzo[d][1,3]dioxol-5-yl)-N-phenylacetamide (**Ib**) has an IR (FTIR/FTNIR-ATR) peak at 1656.92 cm^−1^ for the amide carbonyl (C=O). The ^1^H NMR (500 MHz, DMSO) is δ 10.12 (s, 1H), 7.71–7.59 (m, 2H), 7.41–7.29 (m, 2H), 7.08 (tt, *J* = 7.3, 1.2 Hz, 1H), 7.01–6.80 (m, 3H), 6.03 (d, *J* = 1.1 Hz, 2H), 3.59 (s, 2H), and ^13^C NMR (126 MHz, DMSO) is δ 169.65, 147.61, 146.38, 139.69, 130.07, 129.16, 129.13, 123.65, 122.57, 119.57, 109.99, 108.53, 101.26, 43.36. HRMS (*m*/*z*): [M + H] + was calculated for C_15_H_14_NO_3_ as 256.0974, with a value of 256.0794.

**2-(benzo[d][1,3]dioxol-5-yl)-N-(2,5-dimethoxyphenyl)acetamide (Ic)**

For 2-(benzo[d][1,3]dioxol-5-yl)-N-(2,5-dimethoxyphenyl)acetamide (**Ic**), the IR (FTIR/FTNIR-ATR) shows an amide carbonyl (C=O) at 1572.68 cm^−1^. The ^1^H NMR (500 MHz, DMSO) is δ 9.17 (s, 1H), 7.75 (d, *J* = 3.1 Hz, 1H), 7.04–6.95 (m, 2H), 6.92 (d, *J* = 7.9 Hz, 1H), 6.85 (dd, *J* = 8.1, 1.7 Hz, 1H), 6.66 (dd, *J* = 8.9, 3.1 Hz, 1H), 6.04 (s, 2H), 3.82 (s, 3H), 3.71 (d, *J* = 4.9 Hz, 5H), and ^13^C NMR (126 MHz, DMSO) is δ 169.94, 153.39, 147.63, 146.39, 143.79, 130.09, 128.66, 122.76, 112.34, 110.12, 108.57, 108.49, 108.24, 101.26, 56.76, 55.78, 43.07. HRMS (*m*/*z*): [M + H] + was calculated for C_17_H_18_NO_5_ as 316.1185, which was found to be 316.1185.

**N-phenylbenzo[d][1,3]dioxole-5-carboxamide (IIa)**

In the case of N-phenylbenzo[d][1,3]dioxole-5-carboxamide (**IIa**), the IR (FTIR/FTNIR-ATR) reveals an amide carbonyl (C=O) at 1649.39 cm^−1^. The ^1^H NMR (500 MHz, DMSO) shows δ 10.10 (s, 1H), 7.85–7.76 (m, 2H), 7.63 (dd, *J* = 8.2, 1.4 Hz, 1H), 7.57 (d, *J* = 1.8 Hz, 1H), 7.44–7.35 (m, 2H), 7.19–7.06 (m, 2H), 6.19 (d, *J* = 1.1 Hz, 2H), and the ^13^C NMR (126 MHz, DMSO) is δ 164.95, 150.50, 147.84, 139.70, 129.22, 129.02, 123.98, 123.30, 120.83, 108.39, 108.19, 102.27. HRMS (*m*/*z*): [M + H] + was calculated for C_14_H_12_NO_3_ as 242.0817, with a value of 242.0814.

**N-(3,4,5-trimethoxyphenyl)benzo[d][1,3]dioxole-5-carboxamide (IIb)**

For N-(3,4,5-trimethoxyphenyl)benzo[d][1,3]dioxole-5-carboxamide (**IIb**), the IR (FTIR/FTNIR-ATR) analysis demonstrates an amide carbonyl (C=O) at 1666.79 cm^−1^. The ^1^H NMR (500 MHz, DMSO) is δ 10.13 (s, 1H), 7.70–7.54 (m, 2H), 7.06 (tt, *J* = 7.3, 1.2 Hz, 1H), 6.88 (s, 2H), 6.79 (dd, *J* = 8.9, 3.0 Hz, 1H), 6.73–6.62 (m, 1H), 6.06 (d, *J* = 1.1 Hz, 2H), 3.74 (s, 3H), 3.72 (s, 3H), 3.66 (s, 3H), and the ^13^C NMR (126 MHz, DMSO) is δ 164.91, 148.78, 147.84, 142.48, 139.57, 130.49, 122.99, 122.23, 121.96, 109.48, 108.39, 108.26, 101.32, 56.76, 56.73, 56.72, 40.75. HRMS (*m*/*z*): [M + H] + was calculated for C_17_H_18_NO_6_ as 332.1134, with a value of 332.1134.

**N-(3-(trifluoromethyl)phenyl)benzo[d][1,3]dioxole-5-carboxamide (IIc)**

N-(3-(trifluoromethyl)phenyl)benzo[d][1,3]dioxole-5-carboxamide (**IIc**) exhibits an IR (FTIR/FTNIR-ATR) peak at 1645.96 cm^−1^ for amide carbonyl (C=O). Its ^1^H NMR (500 MHz, DMSO) is δ 10.10 (s, 1H), 7.89–7.81 (m, 1H), 7.78–7.71 (m, 1H), 7.68–7.60 (m, 1H), 7.57–7.45 (m, 1H), 7.44–7.37 (m, 1H), 7.33–7.25 (m, 1H), 7.21–7.13 (m, 1H), 6.15 (d, *J* = 1.1 Hz, 2H), and ^13^C NMR (126 MHz, DMSO) is δ 164.92, 150.51, 147.87, 147.53, 142.48, 131.54, 129.15, 128.85, 123.94, 123.83, 122.75, 120.85, 119.00, 115.33, 108.39, 108.38, 102.27. HRMS (*m*/*z*): [M + H] + was calculated for C_15_H_11_F_3_NO_3_ as 310.0691, and the value found was 310.0696.

**N-(4-(2-methoxyphenoxy)phenyl)benzo[d][1,3]dioxole-5-carboxamide (IId)**

In the case of N-(4-(2-methoxyphenoxy)phenyl)benzo[d][1,3]dioxole-5-carboxamide (**IId**), the IR (FTIR/FTNIR-ATR) reveals an amide carbonyl (C=O) at 1665.74 cm^−1^. The ^1^H NMR (500 MHz, DMSO) is δ 10.14 (s, 1H), 7.73–7.64 (m, 2H), 7.55–7.47 (m, 2H), 7.21 (tt, *J* = 7.6, 1.4 Hz, 1H), 6.95–6.77 (m, 3H), 6.67 (dd, *J* = 8.6, 3.0 Hz, 1H), 6.17 (d, *J* = 1.1 Hz, 2H), 3.79 (s, 3H), 3.64 (d, *J* = 4.5 Hz, 2H), 3.45 (s, 5H), and ^13^C NMR (126 MHz, DMSO) is δ 165.09, 148.96, 147.84, 139.68, 131.09, 129.28, 129.15, 122.96, 122.41, 120.29, 109.34, 108.19, 106.24, 102.29, 56.75, 54.62, 40.77. HRMS (*m*/*z*): [M + H] + was calculated for C_21_H_18_NO_5_ as 364.1185, and the value found was 364.1183.

**N-(3,5-dimethoxyphenyl)benzo[d][1,3]dioxole-5-carboxamide (IIe)**

N-(3,5-dimethoxyphenyl)benzo[d][1,3]dioxole-5-carboxamide (**IIe**) shows IR (FTIR/FTNIR-ATR) absorption at 1645.47 cm^−1^ for amide carbonyl (C=O). The ^1^H NMR (500 MHz, DMSO) shows δ 10.12 (s, 1H), 7.71–7.58 (m, 2H), 7.20 (tt, *J* = 7.3, 1.2 Hz, 1H), 7.06 (s, 2H), 6.82–6.72 (m, 1H), 6.66–6.57 (m, 1H), 6.06 (d, *J* = 1.1 Hz, 2H), 3.74 (s, 3H), 3.69 (s, 3H), and the ^13^C NMR (126 MHz, DMSO) is δ 164.93, 148.82, 147.87, 142.54, 142.48, 139.53, 130.59, 122.97, 122.27, 121.98, 109.51, 108.19, 108.18, 101.32, 56.76, 56.72. HRMS (*m*/*z*): [M + H] + was calculated for C_16_H_16_NO_5_ as 302.1028, and the value found was 302.1028.

### 2.3. α-Amylase Inhibitory Evaluation

The method for assessing α-amylase inhibition was modeled after the protocol delineated by Wickramaratne et al. [38], though certain modifications were implemented. The experiment was conducted using the 3,5-dinitrosalicylic acid (DNSA) approach. Buffer solutions were prepared, containing 20 mm of both sodium phosphate monobasic and sodium phosphate dibasic, and were augmented with 6.7 mm sodium chloride (NaH_2_PO_4_ and Na_2_HPO_4_, each at 6.7 mm NaCl, pH 6.9). A beaker was filled partially with NaH_2_PO_4_ and NaCl solution and stirred magnetically, and the pH was meticulously calibrated using a specialized electrode. The desired pH of 6.9 was achieved by adding more NaH_2_PO_4_ and NaCl to the solution.

The stock solution consisted of synthesized molecules at a 1 mg/mL concentration, harmoniously blended with no less than 10% DMSO in a 1:100 dilution. This mixture was then united with a buffer composed of Na_2_HPO_4_/NaH_2_PO_4_ (0.02 M) and NaCl (0.006 M), maintaining a pH of 6.9. From this, working solutions were created at concentrations ranging from 0.1 to 100 µM. These comprised 0.01, 0.1, 0.5, 1, and 5 mL of the synthesized molecules diluted with the pH-adjusted buffer and brought to a total volume of 10 mL using VF (10 mL). Acarbose was employed as a reference standard and subjected to an identical preparation procedure as that used for the synthesized compounds.

A solution hosting α-amylase at a concentration of 2 units/mL was meticulously crafted, born from the dissolution of 12.5 mg of amylase within a precise quantity of 10% DMSO. This transformative amalgam journeyed to attain a final volume of 100 mL, seamlessly infused with the phosphate buffer discussed earlier, harmonizing within a 100 mL volumetric flask (VF). Meanwhile, the starch solution, poised at a concentration of 1% (*w*/*v*), saw the suspension of 1000 mg of starch in 100 mL of distilled water, orchestrated with finesse within a 100 mL VF. This aqueous symphony unfolded in a temperate embrace of 37 °C within a water bath, occasionally stirring to deter any starch precipitation inklings.

The overture of this reaction was orchestrated by DNSA—a reactive virtuoso that commences its dance upon encountering reducing sugars, conjuring forth 3-amino-5-nitro salicylic acid, a spectral charmer at the wavelength of around 540 nm. The creation of DNSA itself, an artful process, saw the dissolution of 12 g of sodium potassium tartrate tetrahydrate within 8.0 mL of 2 M NaOH, further entwined with 20 mL of the enchanted 96 mm 3.5-dinitrosalicylic acid solution. Thus, the stage was set for this chemical ballet, where reactions waltzed, and absorbance painted a canvas of molecular magic.

The experimental methodology encompassed a deliberate fusion of components, initiating with the gentle agitation of 200 μL of α-amylase solution (2 units/mL) alongside an equal volume of the respective volatile oil working solutions. This harmonious blend underwent a meticulous incubation period at 37 °C, extending over 10 min. Following this orchestrated phase, an additional infusion of 200 μL of the starch solution graced each test tube, orchestrating a subsequent 3 min interlude at the same 37 °C tempo.

The climax of this reaction narrative was unveiled as 200 μL of DNSA entered the stage, promptly followed by a vigorous boiling performance for 10 min, orchestrated at the harmonious temperature range of 85 to 90 °C. The denouement of this experimental symphony saw the composition cooling to room temperature, transitioning into a delicate dilution with 5 mL of distilled water. In a final crescendo, the resounding absorbance at 540 nm resounded as it was masterfully measured utilizing the UV-Visible spectrophotometer.

In order to produce the initial state, the blank canvas was used to substitute the synthesized chemicals, adding 200 μL of buffer. This created a calm and subdued introduction before the commencement of the lively collective. Amidst this orchestration, acarbose assumed the role of a leading virtuoso, serving as the positive control sample. This captivating performance culminated with quantifying enzyme inhibitory activity expressed as a percentage of inhibition. The elusive IC_50_ value for the compounds gracefully materialized through a calculated formula, lending an eloquent conclusion to this experimental symphony [39].

α-amylase inhibitory (%) = [ABSblank − ABStest]/[ABSblank]) × 100%

### 2.4. In Vivo Evaluation of the Antidiabetic Effect 

#### 2.4.1. Animals

The mice-related investigations were meticulously conducted, adhering strictly to the protocols outlined by the Association for Assessment and Accreditation of Laboratory Animal Care International. The ethical underpinning of these experiments was formally acknowledged, having secured the approval of the Institutional Review Board of the Animal House and Use Committee at An-Najah National University (approval number: Med. June. 2023/9).

The study’s protagonists consisted of 18 healthy adult mice gracing the stage with a weight symphony ranging from 17 to 27 g. These cherished participants were thoughtfully accommodated in groups of three within the hospitable confines of the animal facility. In an overture of preparation, the mice were granted 7 days to acquaint themselves with the ambiance of the animal facility, enveloped within the embrace of controlled temperature conditions, a harmonious 25 ± 2 °C.

The experimental groups included a control group that did not receive a streptozotocin (STZ) injection (group 1) and two diabetes groups that were injected with STZ (groups 2 and 3). In group 3, after the STZ injection, the mice were further administered compound **IIc** via intraperitoneal injection.

#### 2.4.2. Induction of Experimental Diabetes

In the experiment, the mice were subjected to diabetes induction via an intraperitoneal (i.p.) injection of streptozotocin (STZ). The STZ was prepared by dissolving it in 0.1 M cold citrate buffer, maintaining a pH of 4.5. Each mouse was injected with a dose of 40 mg/kg of body weight. In order to ascertain the induction of diabetes, the fasting blood glucose levels of the mice were assessed on the fourth day after the injection of STZ. An increase in the fasting blood glucose level at this point was used as a sign of successful induction of the disease [40]. On the 10th day, following seven injections of STZ, all mice were fasted for 6 h, and a blood glucose test was subsequently performed using a blood sample drawn from the tail vein [41]. 

#### 2.4.3. Drug Administration and Measurement of Blood Glucose Levels 

In this research, the fasting blood glucose levels for all groups were measured on the first day before the induction of diabetes using a streptozotocin (STZ) injection. The development of diabetes was then verified by monitoring fasting blood glucose levels on both the 4th and 10th days following the injection. At the end of the diabetes induction period, the mice in group 3 that were diagnosed with diabetes were treated daily with an i.p. injection of compound **IIc** at a dose of 10 mg/kg for 5 consequent days. Meanwhile, the remaining groups, including group 1 (non-diabetic) and group 2 (diabetic), were maintained as control groups.

### 2.5. Cell Culture and MTS Assay

Cultivation of the cancer cell lines MCF-7, Hep3B, HepG2, and Caco-2 HeLa was conducted in RPMI 1640 medium, whereas LX-2, B16-F1, and HEK-293T cells were grown in DMEM medium. These cells were incubated at 37 °C in a 5% CO_2_ atmosphere within a humid setting. In a 96-well plate, 1000 cells were seeded in each well. Following a 24 h incubation, they were exposed to various concentrations of **Ia-IIe** compounds (300, 100, 50, and 10 µM) for 72 h. The Cell-Tilter 96^®^ (Promega Corporation, Madison, WI, USA) Aqueous One Solution Cell Proliferation (MTS) bioassay was utilized to determine the cell viability post-culture. For this assay, 90 µL of medium and 10 µL of MTS solution were added to every well, and the plates were then placed in a 37 °C environment for 2 to 4 h. The absorbance was subsequently assessed at a wavelength of 490 nm using a UV–Vis spectrophotometer [42].

## 3. Results

### 3.1. Chemistry

Following Scheme 1, a series of derivatives, **Ia-Ic** and **IIa-IIe**, were synthesized, including benzodioxole carboxamide compounds. The process was commenced by dissolving 2-(benzo[d][1,3]dioxol-5-yl)acetic acid or benzo[d][1,3]dioxole-5-carboxylic acids in dichloromethane (DCM). Subsequently, a mixture of DMAP and EDCI was introduced and thoroughly combined under an argon gas environment. The corresponding aniline derivatives were added after a 30 min incubation period for each experiment. The ensuing reaction was stirred for 48 h. After this reaction period, extraction was carried out using 32% HCl solution, followed by treatment with anhydrous sodium sulfate, and the process was concluded with filtration [43].

### 3.2. MicroED Characterization

All compounds were characterized using common methods, including FT-IR, HRMS, ^1^H-NMR, and ^13^C-NMR, with accurate values and spectra. Moreover, the most active compound **IIc**, beside our control compound **IIe**, was characterized using the MicroED method. MicroED, one of the 3D electron diffraction (3D ED) methods, is considered a valuable tool for characterizing small molecules with limited availability or crystallographic challenges, especially when only microcrystals are available. It offers a powerful alternative to traditional X-ray crystallography, allowing researchers to determine the atomic structure of challenging samples using a transmission electron microscope. Here, the microED method was applied for the investigated compounds because it was impossible to obtain crystals large enough for X-ray diffraction structure determination. 

**Figure 2:**
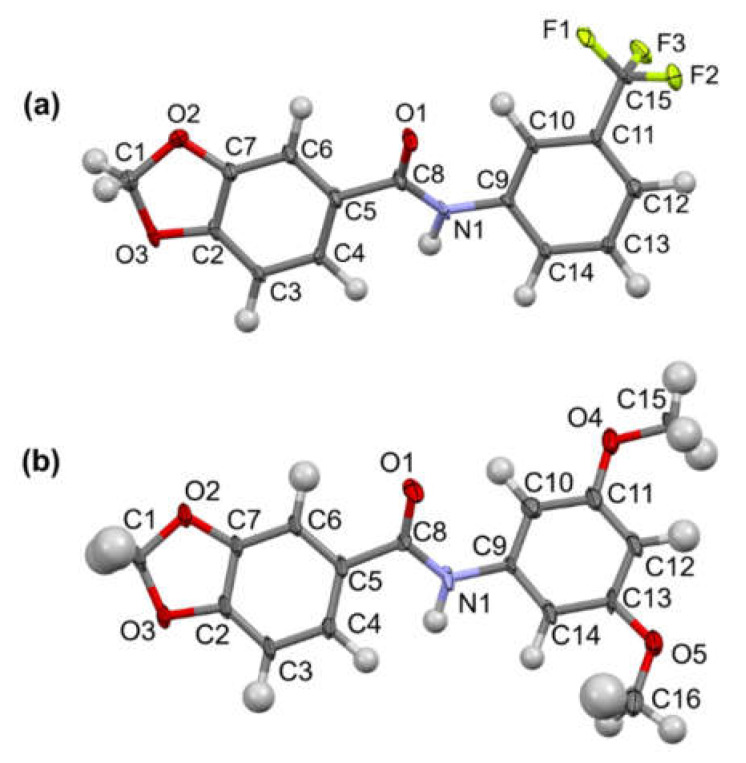
Thermal ellipsoid views with 50% probability level for (**a**) **IIc** and (**b**) **IIe** with the labels of atoms.

**Figure 3:**
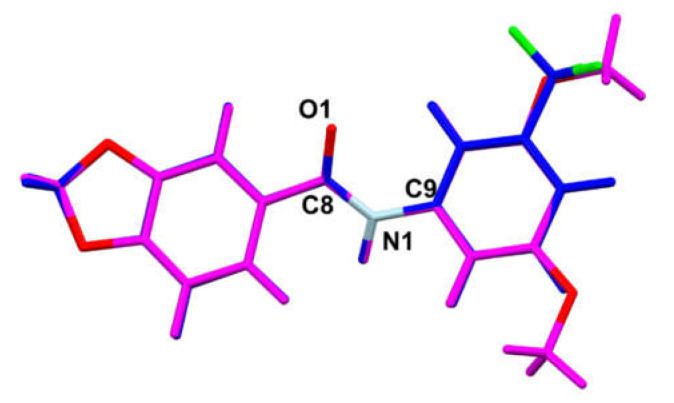
Structure overlay diagrams of the compound **IIc** (blue) and **IIe** (magenta).

The electron diffraction data for **IIc** and **IIe** were collected on one microcrystal (Figure S1) of each compound. Relatively good-quality and complete datasets were collected (Table S1). Kinematical TAAM refinement led to a crystal structure model of fair quality (Tables S2–S7), with all the ADPs of non-H atoms positively defined, as shown in Figure 2. The microED results confirm the composition and atom connectivity of the synthesized compounds. Moreover, it provided insight into the 3D structure of the compounds. The 3D structure of these compounds was unknown until now to best of our knowledge, and the 3D structures of only two compounds of similar compositions were found in the Cambridge Structural Database (2-[(2H-1,3-benzodioxole-5-carbonyl)amino]phenyl 4-fluorobenzene-1-sulfonate [44] and N-(2-Carbamoylphenyl)-6-nitroso-1,3-benzodioxole-5-carboxamide [45]. The structural overlay of compounds **IIc** and **IIe** for the atoms C8-N1-C9 shows that the conformation of both compounds is the same, as shown in Figure 3. On the basis of only two compounds, it is hard, however, to conclude that the observed conformation is typical of the whole series of the herein investigated compounds and will be maintained in the complex with the targeted protein. The crystallographic data and refinement parameters are summarized in Table S1. The bond lengths, bond angles, and torsion angles of compounds **IIc** and **IIe** are listed in Tables S2–S7. 

### 3.3. In Vitro α-Amylase Inhibitory Effects 

The α-amylase inhibitory effects of the synthesized derivatives were used to evaluate their in vitro antidiabetic efficacy. As a positive control, acarbose was used. To compute the IC_50_ values of these substances, the inhibitory power of a series of five concentrations of 0.1, 1, 10, 50, and 100 µM were utilized (Table 1).

The compounds were studied in this work for their interaction with alpha-amylase enzyme, with particular attention paid to the binding region of the protein structure represented by PDB ID 4W93. **IIa** had an IC_50_ value of 0.85 µM, and **IIc** had an IC_50_ value of 0.683 µM. These compounds demonstrated remarkable effectiveness against alpha-amylase, which could be attributed to certain structural properties. The compounds with higher potency demonstrated a variety of interactions when assessing their potential binding interactions within the binding site of PDB ID 4W93. Hydrogen bonding, pi–pi stacking, and hydrophobic interactions could all be involved. According to our previous work on similar compounds, these binding interactions are suggested to be present between the benzodioxol ring, phenyl-substituted groups, and the amino acids in the enzyme [28]. In the past, certain naturally occurring substances have been documented for their ability to inhibit human pancreatic α-amylase (HPA). These substances primarily include saccharides like acarbose [46], as well as flavonoid compounds such as myricetin and montbretin A (MbA). Montbretin A, specifically, is a glycosylated flavonol linked to a caffeic acid component [47]. The crystallographic analysis of the MbA-HPA complex, available under PDB id: 4w93 with a resolution of 1.35 Å, revealed a significant reliance on hydrogen bond interactions between residues of HPA and the hydroxyl groups present in MbA. Notably, the X-ray structure displays a distinctive bound conformation of MbA characterized by intermolecular pi–pi stacking interactions between two aromatic rings, namely, aromatic ring A from the flavonoid core and the aromatic ring associated with the caffeic moiety. These hydrogen bonds are established either directly or mediated by water molecules, linking the bound ligand to specific protein residues. These include the catalytic residues E233 and D197, as well as other pertinent residues, such as the I235, H201, H305, K200, E240, H101, Q63, N298, D356, R159, and D300 moieties [47]. 

**Figure 4:**
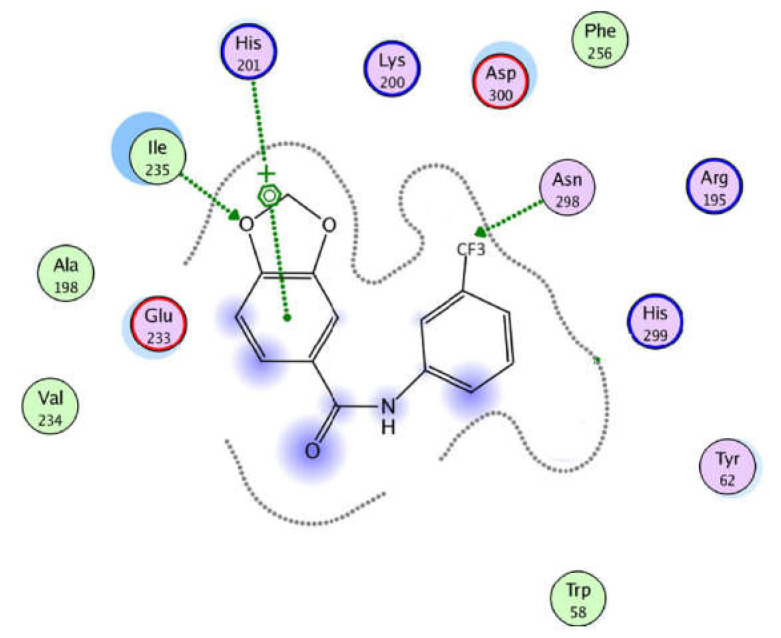
Possible binding interactions between compound **IIc** and Asn298 (orthogonal multipolar interactions), His201 (charge-π interactions), and Ile235 (hydrogen bonds).

Similarly, acarbose has been observed to engage in hydrogen bond interactions with analogous residues of pig pancreatic α-amylase, documented under PDB id 1OSE. These residues encompass D197, E233, H101, H201, K200, Y62, Y151, and others [48]. Furthermore, prior publications have firmly established a pair of critical catalytic residues essential for α-amylase function: an aspartate residue serving as the nucleophile and a glutamate residue functioning as the acid–base catalyst [49]. The combination of a phenyl and trifluoromethyl group in compound **IIc** could allow orthogonal multipolar interactions, with backbone carbonyl groups and side chains of the amino acid Asn298 (Figure 4) forming hydrogen bonds with the residue Ile235 amino acid, and may allow charge-π interactions with His201.

It is worth noting that the specific binding interactions and their contributions to the observed activities need further investigation, which could include experimental research or computer simulations. The data presented here provide a preliminary knowledge of the compounds’ potency, structural characteristics, potential interactions in PDB ID 4W93, and alpha-amylase inhibition.

### 3.4. In Vivo Hypoglycemic Effect of Compound IIc on Mice with STZ-Induced Diabetes

**Figure 5:**
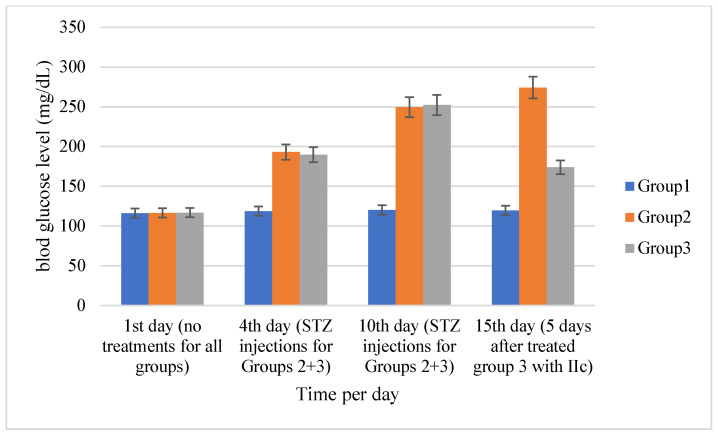
Fasting serum glucose concentration on the 1st day without any treatment, the 4th and 10th day with STZ for groups 2 + 3, and the 15th day after treatment for group 3 with the compound **IIc**. Data are shown as mean ± SD (*n* = 6); *p*-value < 0.05 (group 1: negative control mice without any treatment; group 2: positive control, mice with STZ treatment; group 3: mice treated with STZ + **IIc**).

Based on the outcomes derived from the in vitro assessment of α-amylase inhibitory effects of the synthesized benzodioxol derivatives, the compound with the greatest potency, **IIc** (N-(3-(trifluoromethyl)phenyl)benzo[d][1,3]dioxole-5-carboxamide), was chosen as a contender for in vivo hypoglycemic exploration. This investigative endeavor aimed to unveil the impact of **IIc** on blood glucose levels in STZ-induced diabetic mice. The findings of this study resoundingly illuminate that **IIc** displays noteworthy hypoglycemic prowess within STZ-diabetic mice, mirroring the substantial effect observed in the control group (untreated mice), as vividly illustrated in Figure 5.

The findings indicate that the initial fasting blood glucose levels exhibited no significant differences across all groups. However, after intraperitoneal administration of STZ (40 mg/kg), a notable elevation in glucose blood concentration was observed as compared to control group 1, as illustrated in Figure 5. Upon administering five doses of **IIc** (10 mg/kg) to group 3, a significant reduction in glucose concentration was observed, bringing it down to 173.8 mg/dL. This decrease was particularly pronounced when contrasted with the untreated diabetes group 2, which displayed a glucose concentration of 274.25 mg/dL.

### 3.5. Cytotoxicity Results

**Figure 6:**
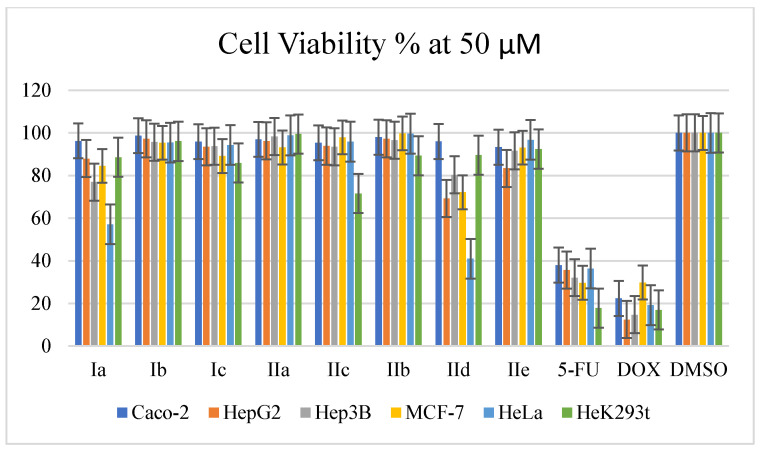
Cell viability values at 50 µM for various cell lines of the benzodioxole derivatives in comparison with positive and negative controls.

Based on the IC_50_ values presented in Table 2 and the cell viability percentage at 50 µM (Figure 6) for various cancer cell lines, compound **IId** (N-(4-(2-methoxyphenoxy)phenyl)benzo[d][1,3]dioxole-5-carboxamide) showed significant anticancer activity when compared to the other compounds. It was clear that this compound showed lower cell viability for the HeLa and HepG2 cancer cell lines, as presented in Figure 6, and this compound’s IC_50_ values were in range 26.59–65.16 µM for all cell lines except Caco-2 cancer cell lines. The presence of a phenoxy group in **IId** may contribute to its anticancer effect. Phenoxy groups have been linked to cytotoxicity and have been used in the development of anticancer drugs. 

## 4. Conclusions

This study underscores the potential of benzodioxol carboxamide derivatives as multifaceted agents with diverse therapeutic applications. A series of compounds were successfully developed through a meticulous synthesis and thorough characterization process, laying the groundwork for further investigation. The application of advanced analytical techniques, including HRMS, ^1^H-, and ^13^C-NMR, and the innovative utilization of MicroED ensured a comprehensive understanding of the synthesized compounds. The incorporation of MicroED provided valuable structural insights and demonstrated the significance of pushing the boundaries of characterization methodologies. The in vitro assessment of the α-amylase enzyme revealed intriguing leads, with the compounds **IIa** and **IIc** emerging as particularly promising candidates. Their potent activity against the target enzyme and the favorable safety profile observed for normal cells indicate a strategic avenue for drug development. The in vivo evaluation of compound **IIc** in a diabetes model provides valuable insights into its antidiabetic properties. The observed reduction in blood glucose levels highlights its prospective role as an antidiabetic agent, warranting continued exploration. 

In summary, this research expands our understanding of benzodioxol derivatives and underscores the importance of innovative characterization techniques like MicroED. The nuanced approach of combining in vitro and in vivo studies enriches the depth of this exploration. These findings set the stage for future studies that could harness the unique attributes of these compounds for the development of innovative treatments targeting diabetes and cancer.

## Figures and Tables

**Table 1:** Chemical structures of the assessed compounds, along with their corresponding IC_50_ values concerning α-amylase inhibition and the Hek293t normal cell line. These bolded values belong to the most potent compound.

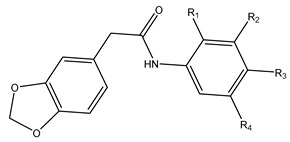 St. I				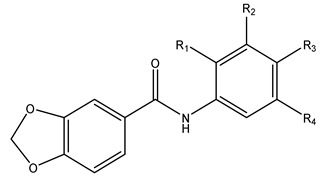 St. II			
Compound	St.	R_1_	R_2_	R_3_	R_4_	IC_50_ µM	
						α-Amylase	Hek293t
**Ia**	**I**	O-CH_3_	H	Cl	O-CH_3_	16.77 ± 1.55	>300
**Ib**	**I**	H	H	H	H	8.44 ± 1.01	269.23 ± 1.73
**Ic**	**I**	O-CH_3_	H	H	O-CH_3_	16.40 ± 1.58	206.64 ± 2.59
**IIa**	**II**	H	H	H	H	0.85 ± 0.18	>300
**IIb**	**II**	H	O-CH_3_	O-CH_3_	O-CH_3_	15.30 ± 1.07	>300
**IIc**	**II**	**H**	**CF_3_**	**H**	**H**	**0.68 ± 0.25**	**155.87 ± 2.98**
**IId**	**II**	H	H	2-OCH_3_Ph	H	5.76 ± 1.92	>300
**IIe**	**II**	H	O-CH_3_	H	O-CH_3_	14.97 ± 3.52 [21]	>300
Acarbose						1.55 ± 0.85	-
DOX						-	0.038 ± 0.02

**Table 2:** The IC_50_ values of the benzodioxole derivatives in comparison with DOX and 5-Fu for various cell liens.

	IC_50_ µM					
	Caco-2	HepG2	Hep3B	MCF-7	HeLa	LX-2
**Ia**	>300	169.44 ± 2.16	196.52 ± 1.99	123.58 ± 2.73	43.65 ± 2.97	70.62 ± 2.45
**Ib**	>300	>300	>300	>300	>300	280.61 ± 2.33
**Ic**	>300	>300	>300	>300	>300	208.87 ± 2.20
**IIa**	>300	>300	>300	>300	>300	>300
**IIb**	>300	>300	>300	>300	>300	96.60 ± 2.70
**IIc**	>300	>300	>300	>300	>300	>300
**IId**	>300	55.33 ± 2.72	65.16 ± 2.53	55.7592 ± 2.51	26.59 ± 2.41	31.43 ± 2.71
**IIe**	>300	>300	>300	>300	>300	183.43 ± 2.00
5-FU	4.75 ± 1.71	4.07 ± 1.42	5.11 ± 1.67	6.51 ± 1.82	7.79 ± 1.60	0.178 ± 0.11
DOX	0.024 ± 0.001	<0.01	0.025 ± 0.005	0.053 ± 0.009	<0.01	0.21 ± 0.12

(*p*-value < 0.05).

## Data Availability

This published article and its Supplementary File include all data generated or analyzed during this study.

## Supplementary Materials

The following supporting information can be downloaded at: https://www.mdpi.com/article/10.3390/biom13101486/s1, Figure S1: TEM images of crystals (a) **IIc** (SSA-11) and (b) **IIe** (SSA-15); Figure S2: NMR Spectrum of (**Ia**) SSA-2; Figure S3: NMR Spectrum of (**Ib**) SSA-3; Figure S4: NMR Spectrum of (**Ic**) SSA-5; Figure S5: NMR Spectrum of (**IIa**) SSA-10; Figure S6: NMR Spectrum of (**IIc**) SSA-11; Figure S7: NMR Spectrum of (**IIb**) SSA-12; Figure S8: NMR Spectrum of (**IId**) SSA-13; Figure S9: NMR Spectrum of (**IIe**) SSA-15; Table S1: Summary of the data collection, reduction, and refinement statistics of the compounds; Table S2: Bond lengths for **IIc** (SSA-11); Table S3: Bond angles for **IIc** (SSA-11); Table S4: Torsion angles for **IIc** (SSA-11); Table S5: Bond lengths for **IIe** (SSA-15); Table S6: Bond angles for **IIe** (SSA-15); Table S7: Torsion angles for **IIe** (SSA-15).
