# Supplementary material for: Characterization and Investigation of Novel Benzodioxol Derivatives as Antidiabetic Agents: An In Vitro and In Vivo Study in an Animal Model
Source: Biomolecules. 2023 Oct 6;13(10):1486. doi: 10.3390/biom13101486 (PMC10604990; doi:10.3390/biom13101486)

Supplementary Material

Characterization and Investigation of Novel Benzodioxol Derivatives as Anti-diabetic agents: An *In vitro* and *In Vivo* study in Animal Model

Mohammed Hawash[1,*], Derar Al-Smadi[2], Anil Kumar[3], Barbara Olech[3,4], Paulina Maria Dominiak[3], Nidal Jaradat[1], Sarah Antari[1], Sarah Mohammed[1], Ala'a Nasasrh[1], Murad Abualhasan[1], Ahmed Musa[5], Shorooq Suboh[5], İrfan Çapan[6,7], Mohammad Qneibi[5], Hiba Natsheh[1,*]

[1] Department of Pharmacy, Faculty of Medicine and Health Sciences, An-Najah National University, Nablus, Palestine.
[2] Department of Chemistry, Faculty of Sciences, An-Najah National University, Nablus, Palestine.
[3] Biological and Chemical Research Centre, Department of Chemistry, University of Warsaw, ul. Żwirki i Wigury 101, 02-089 Warsaw, Poland
[4] Centre of New Technologies, University of Warsaw, ul. S. Banacha 2c, 02-097 Warsaw, Poland
[5] Department of Biomedical Sciences, Faculty of Medicine and Health Sciences, An-Najah National University, Nablus, Palestine
[6] Gazi University, Technical Sciences Vocational College, Department of Material and Material Processing Technologies, 06560 Ankara, Türkiye
[7] Gazi University, Basic and Engineering Sciences Central Laboratory Application and Research Center (GUTMAM), 06500, Ankara, Türkiye

[*]**Corresponding author**: **Mohammed Hawash**, Department of Pharmacy, Faculty of Medicine and Health Sciences, An-Najah National University, Nablus, PALESTINE, orcid.org/0000-0001-5640-9700; Phone: +972569939939; Email: mohawash@najah.edu.

**Figures and Tables**	**Pages**

Figure S1: TEM images of crystals (a) IIc (**SSA-11)** and (b) IIe (**SSA-15)…………** **3**

Table S1: Summary of the data collection, reduction, and refinement statistics of the compounds**………………………………………………………………………….** **4**

Table S2: Bond lengths for IIc (**SSA-11) ………………………………………………..** **5**

Table S3: Bond angles for IIc (**SSA-11) ………………………………………………..** **6**

Table S4: Torsion angles for IIc (**SSA-11) ………………………………….………..** **7**

Table S5: Bond lengths for IIe (**SSA-15)……………………………………………….** **8**

Table S6: Bond angles for IIe (**SSA-15)………………………………………………..** **9**

Table S7: Torsion angles for IIe (**SSA-15)…………………………………………..** **10**

Figure S2. NMR Spectrum of (Ia) SSA-2 …………………………………………. **11**

Figure S3. NMR Spectrum of (Ib) SSA-3………………..…………………….. **12**

Figure S4. NMR Spectrum of (Ic) SSA-5………………………………………….. **13**

Figure S5. NMR Spectrum of (IIa) SSA-10…………………………..…….. **14**

Figure S6. NMR Spectrum of (IIc) SSA-11…………………………..…….. **15**

Figure S7. NMR Spectrum of (IIb) SSA-12…………………………..…….. **16**

Figure S8. NMR Spectrum of (IId) SSA-13…………………………..…….. **17**

Figure S9. NMR Spectrum of (IIe) SSA-15…………………………..…….. **18**

Figure S1: TEM images of crystals (a) IIa **SSA-11** and (b) IIe **SSA-15.**

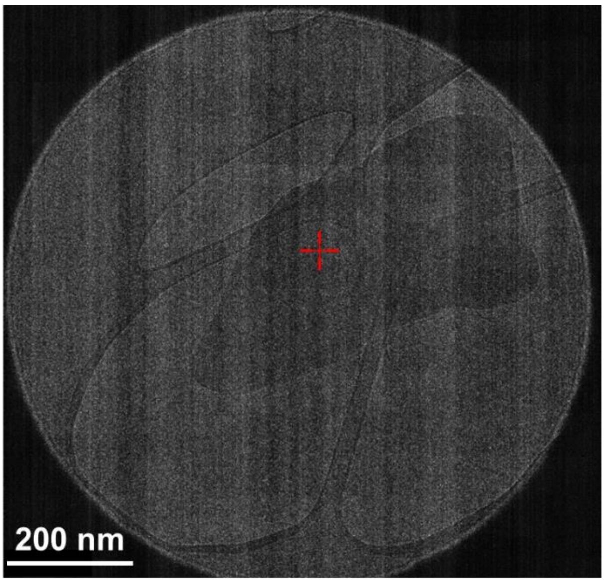

(a)

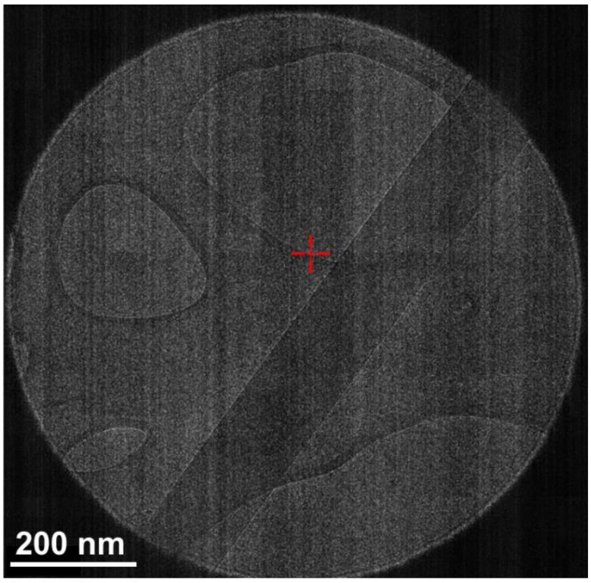

(b)

Table S1: Summary of the data collection, reduction, and refinement statistics of the compounds.

Compound ID	SSA-11 **IIc**	SSA-15 **IIe**
Chemical Formula	$C_{15} H_{10} F_3 N O_3$	$C_{16} H_{15} N_2 O_5$
Oscillation per frame (^{o})	0.3	0.4
Detector distance (mm)	644.72	
Temperature (^{o}C)	-192	
Accelerating voltage (kV)	200	
Wavelength (Å)	0.02508	
Space group	*Cc*	*C*2/*c*
Unit cell *a, b, c* (Å)	7.1, 6.8, 24.3	28.3, 4.9, 20.4
Angles *α, β, γ* (^{o})	90.0, 91.9, 90.0	90.0, 114.8, 90.0
Volume (Å^3)	1172.56	2567.98
Z/Z'	4/1	8/1
Resolution (Å)	0.70	0.80
Total reflections	4279	5858
Unique reflections	2619	2123
Completeness (%)	74.3	79.9
Mean *I/σ*	5.9	6.1
R_{int} (%)	10.44	14.38
Data/restraints/parameters	2619/2/188	2123/0/205
R_1, wR_2 [I>=2σ (I)]	0.1060, 0.2691	0.1419, 0.3498
R_1, wR_2 [all data]	0.1349, 0.2939	0.2187, 0.4060
Goodness of fit (*S*)	0.989	1.259
Peak/Hole (Å^{-2})	0.171/-0.159	0.215/-0.248

Table S2: Bond lengths for **SSA-11**

Atom	Atom	Length/Å	Atom	Atom	Length/Å
C8	O1	1.219(7)	N1	C9	1.398(6)
C8	N1	1.292(7)	O3	C2	1.331(6)
C8	C5	1.473(7)	O3	C1	1.375(8)
F3	C15	1.309(9)	C9	C10	1.353(7)
F1	C15	1.289(7)	C9	C14	1.347(7)
C4	C5	1.370(7)	C15	C11	1.469(7)
C4	C3	1.370(7)	C7	C2	1.350(7)
F2	C15	1.332(8)	C10	C11	1.379(7)
C6	C7	1.358(7)	C2	C3	1.355(7)
C6	C5	1.361(7)	C13	C12	1.371(7)
O2	C7	1.316(6)	C13	C14	1.370(7)
O2	C1	1.406(7)	C11	C12	1.350(7)

Table S3: Bond angles for **SSA-11**

Atom	Atom	Atom	Angle/°	Atom	Atom	Atom	Angle/°
N1	C8	O1	123.8(5)	C2	C7	C6	121.9(5)
C5	C8	O1	119.6(5)	C2	C7	O2	109.4(5)
C5	C8	N1	116.5(5)	C11	C10	C9	119.4(5)
C3	C4	C5	122.9(5)	C7	C2	O3	110.4(5)
C5	C6	C7	118.1(5)	C3	C2	O3	128.0(5)
C1	O2	C7	105.6(4)	C3	C2	C7	121.6(5)
C9	N1	C8	125.1(5)	C4	C5	C8	122.7(5)
C1	O3	C2	105.2(5)	C6	C5	C8	118.1(5)
C10	C9	N1	122.8(5)	C6	C5	C4	119.2(5)
C14	C9	N1	117.4(4)	C14	C13	C12	120.4(5)
C14	C9	C10	119.8(4)	C2	C3	C4	116.2(5)
F1	C15	F3	107.4(6)	C10	C11	C15	119.4(5)
F2	C15	F3	106.9(5)	C12	C11	C15	118.8(4)
F2	C15	F1	105.4(6)	C12	C11	C10	121.6(5)
C11	C15	F3	113.1(6)	C11	C12	C13	118.1(5)
C11	C15	F1	112.2(5)	C13	C14	C9	120.7(4)
C11	C15	F2	111.4(6)	O3	C1	O2	108.2(4)
O2	C7	C6	128.6(5)				

Table S4: Torsion angles for **SSA-11**

Atom	Atom	Atom	Atom	Angle/°	Atom	Atom	Atom	Atom	Angle/°
C8	N1	C9	C10	-33.1(9)	C6	C7	O2	C1	-171.9(8)
C8	N1	C9	C14	148.6(7)	C6	C7	C2	O3	178.6(7)
C8	C5	C4	C3	175.3(7)	C6	C7	C2	C3	-1.1(9)
C8	C5	C6	C7	-176.3(6)	O2	C7	C2	O3	-0.1(7)
F3	C15	C11	C10	-94.0(6)	O2	C7	C2	C3	-179.9(6)
F3	C15	C11	C12	80.3(7)	O2	C1	O3	C2	10.6(6)
F1	C15	C11	C10	27.7(8)	N1	C9	C10	C11	-177.8(8)
F1	C15	C11	C12	-158.0(7)	N1	C9	C14	C13	179.5(6)
C4	C5	C6	C7	0.9(9)	C9	C10	C11	C15	172.5(7)
C4	C3	C2	O3	-179.4(7)	C9	C10	C11	C12	-1.6(9)
C4	C3	C2	C7	0.3(9)	C9	C14	C13	C12	-1.6(9)
F2	C15	C11	C10	145.5(6)	C15	C11	C12	C13	-173.0(7)
F2	C15	C11	C12	-40.2(8)	C10	C11	C12	C13	1.2(9)

Table S5: Bond lengths for **SSA-15**

Atom	Atom	Length/Å	Atom	Atom	Length/Å
O3	C2	1.343(6)	N1	C8	1.316(6)
O3	C1	1.389(9)	O1	C8	1.193(6)
O5	C13	1.324(9)	C4	C3	1.369(7)
O5	C16	1.399(11)	C6	C7	1.342(7)
C9	N1	1.383(6)	C11	C10	1.368(7)
C9	C10	1.358(9)	C11	C12	1.343(10)
C9	C14	1.361(10)	C3	C2	1.314(9)
C5	C4	1.357(8)	O2	C7	1.314(8)
C5	C6	1.345(8)	O2	C1	1.383(8)
C5	C8	1.448(7)	C7	C2	1.350(9)
O4	C11	1.345(10)	C14	C13	1.360(7)
O4	C15	1.395(7)	C12	C13	1.361(9)

Table S6: Bond angles for **SSA-15**

Atom	Atom	Atom	Angle/°	Atom	Atom	Atom	Angle/°
C1	O3	C2	104.8(5)	C12	C11	C10	123.0(6)
C16	O5	C13	116.9(5)	C2	C3	C4	117.5(6)
C10	C9	N1	121.5(6)	C11	C10	C9	117.6(7)
C14	C9	N1	117.0(6)	C1	O2	C7	106.2(6)
C14	C9	C10	121.5(5)	O2	C7	C6	129.8(6)
C6	C5	C4	119.4(5)	C2	C7	C6	120.2(6)
C8	C5	C4	123.2(5)	C2	C7	O2	110.0(5)
C8	C5	C6	117.3(5)	C13	C14	C9	118.2(6)
C15	O4	C11	115.9(7)	C13	C12	C11	117.3(5)
C8	N1	C9	126.3(5)	C3	C2	O3	128.0(6)
C3	C4	C5	121.2(6)	C7	C2	O3	109.7(6)
C7	C6	C5	119.3(6)	C7	C2	C3	122.3(5)
N1	C8	C5	117.4(4)	C14	C13	O5	123.9(7)
O1	C8	C5	120.7(5)	C12	C13	O5	113.8(5)
O1	C8	N1	121.9(5)	C12	C13	C14	122.3(7)
C10	C11	O4	113.4(6)	O2	C1	O3	108.7(5)
C12	C11	O4	123.4(5)				

Table S7: Torsion angles for **SSA-15**

Atom	Atom	Atom	Atom	Angle/°	Atom	Atom	Atom	Atom	Angle/°
O3	C2	C3	C4	179.4(10)	C9	C14	C13	C12	0.7(10)
O3	C2	C7	C6	178.0(7)	C5	C4	C3	C2	1.3(10)
O3	C2	C7	O2	-0.8(9)	C5	C6	C7	O2	-177.8(8)
O3	C1	O2	C7	-7.4(10)	C5	C6	C7	C2	3.7(10)
O5	C13	C14	C9	-178.8(9)	O4	C11	C12	C13	-177.2(8)
O5	C13	C12	C11	179.9(8)	C4	C3	C2	C7	-0.0(11)
C9	N1	C8	C5	-176.5(10)	C6	C7	O2	C1	-173.6(11)
C9	N1	C8	O1	3.6(12)	C6	C7	C2	C3	-2.5(11)
C9	C10	C11	O4	178.6(7)	C11	C12	C13	C14	0.3(10)
C9	C10	C11	C12	3.3(9)	C3	C2	C7	O2	178.8(10)

FigureS2. 2-(benzo[d][1,3]dioxol-5-yl)-N-(4-chloro-2,5-dimethoxyphenyl)acetamide (Ia)

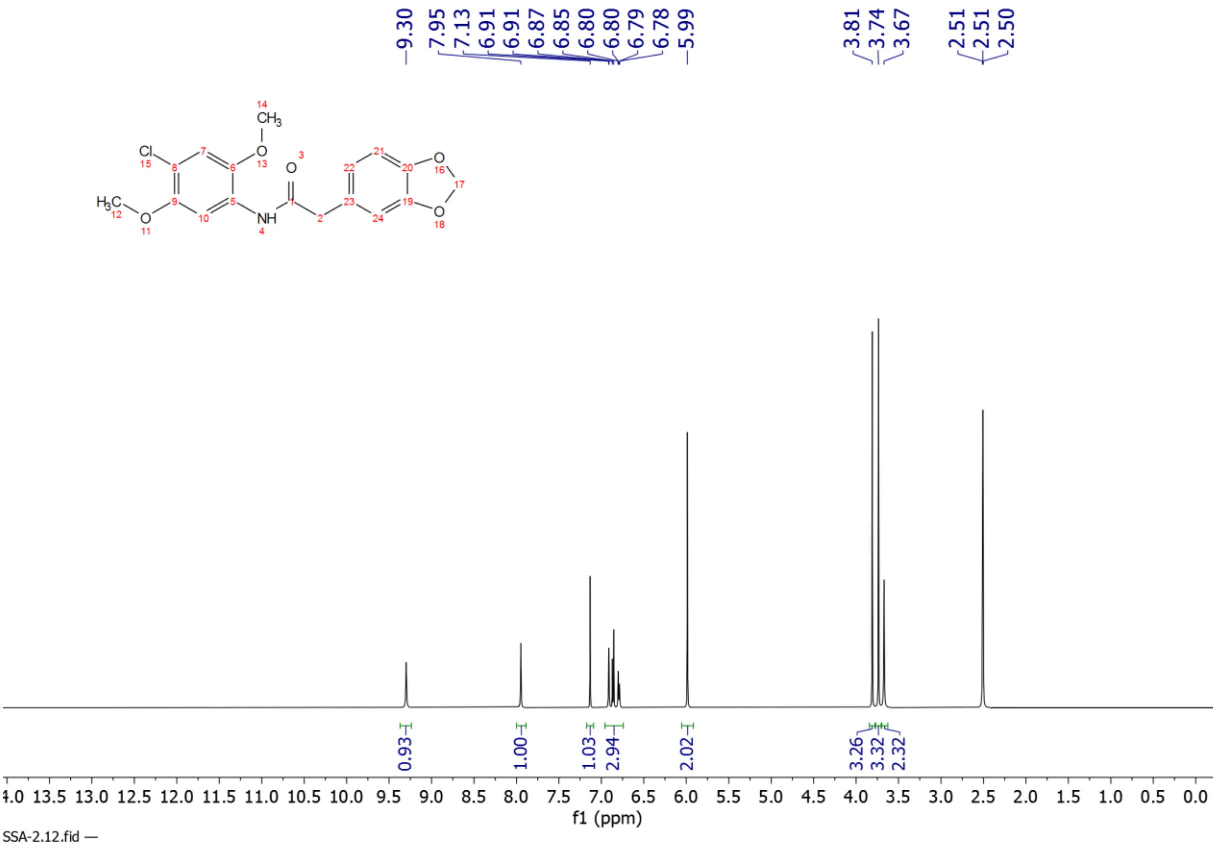

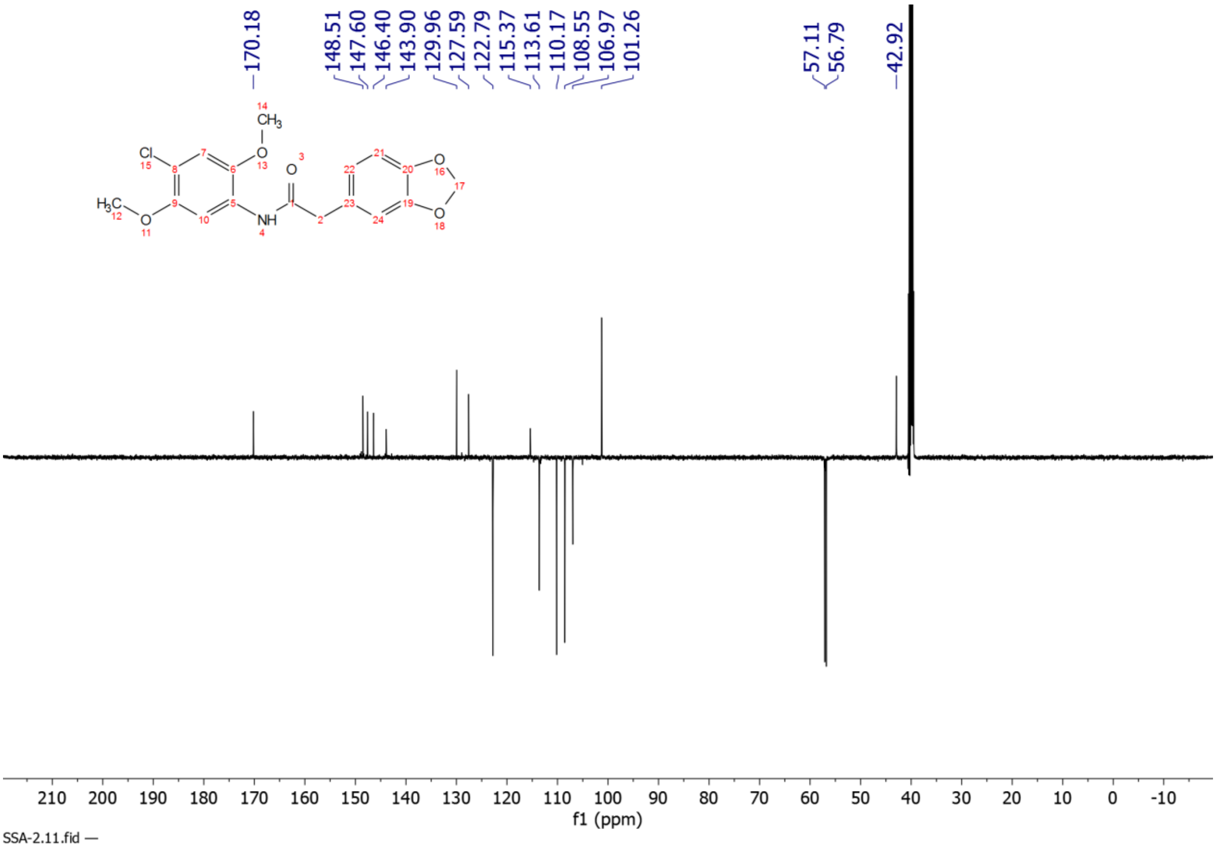

Figure S3. 2-(benzo[d][1,3]dioxol-5-yl)-N-phenylacetamide (Ib) SSA-3

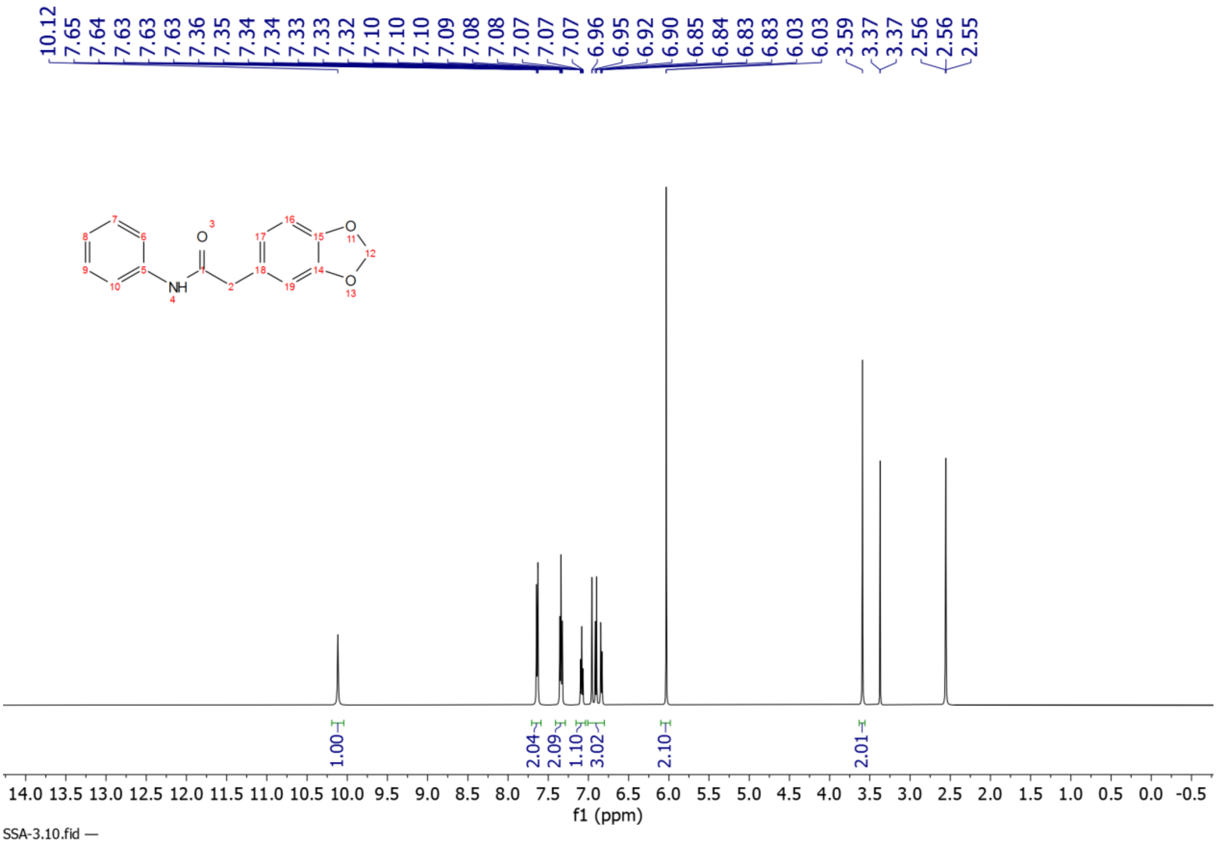

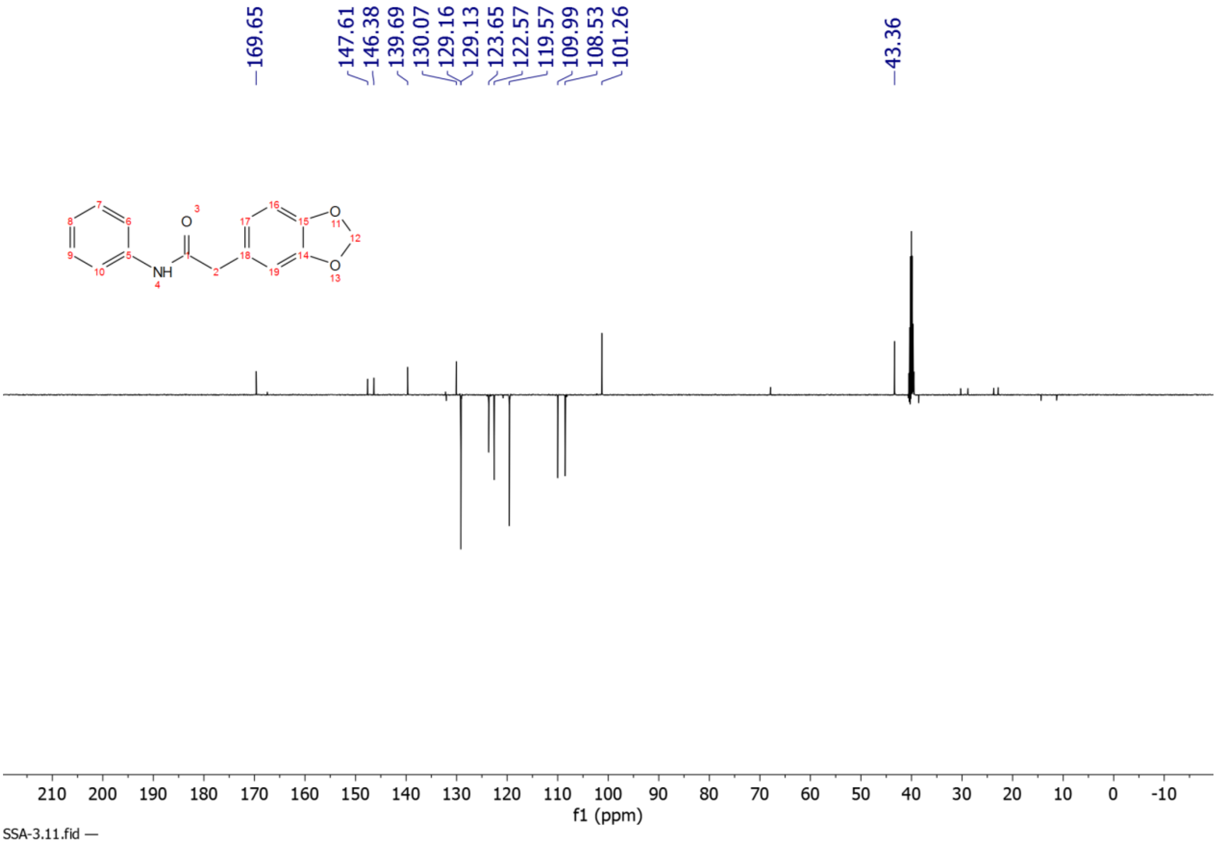

Figure S4. **2-(benzo[d][1,3]dioxol-5-yl)-N-(2,5-dimethoxyphenyl)acetamide (Ic) SSA-5**

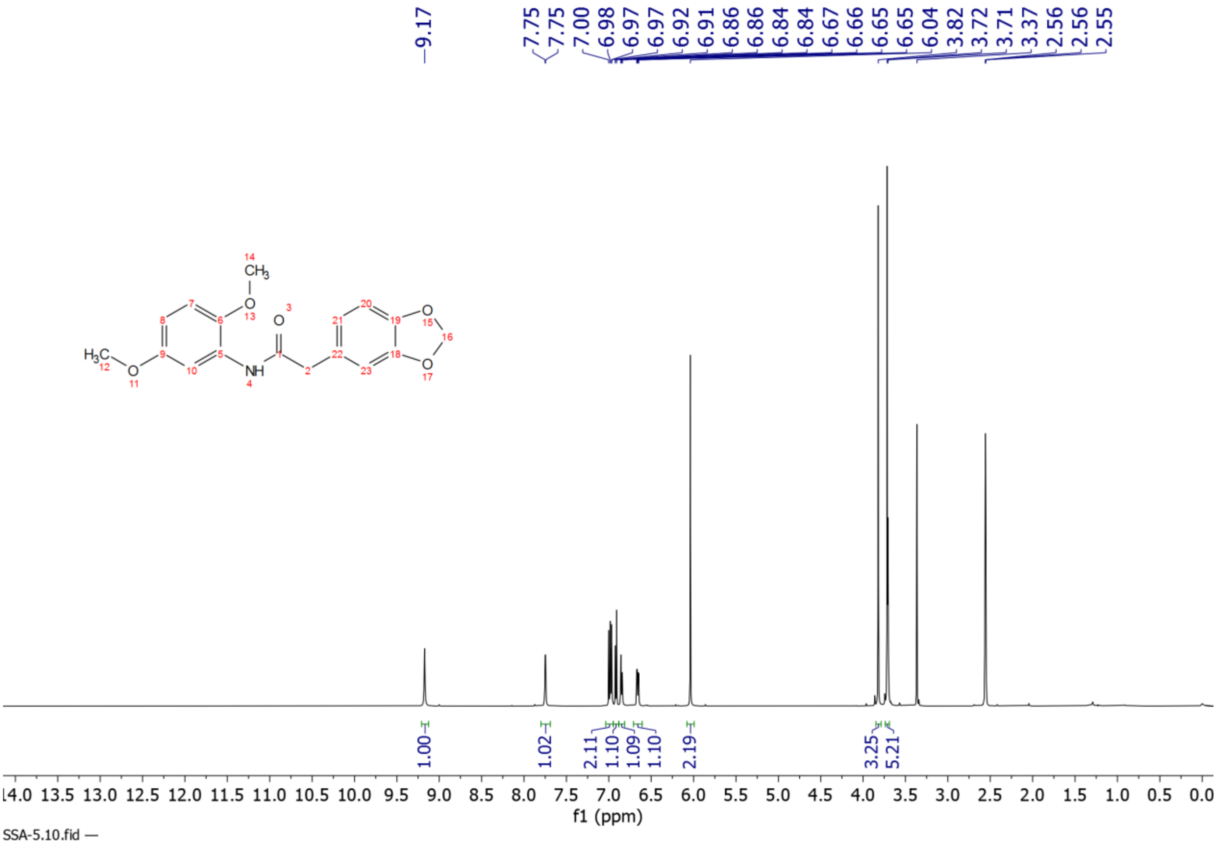

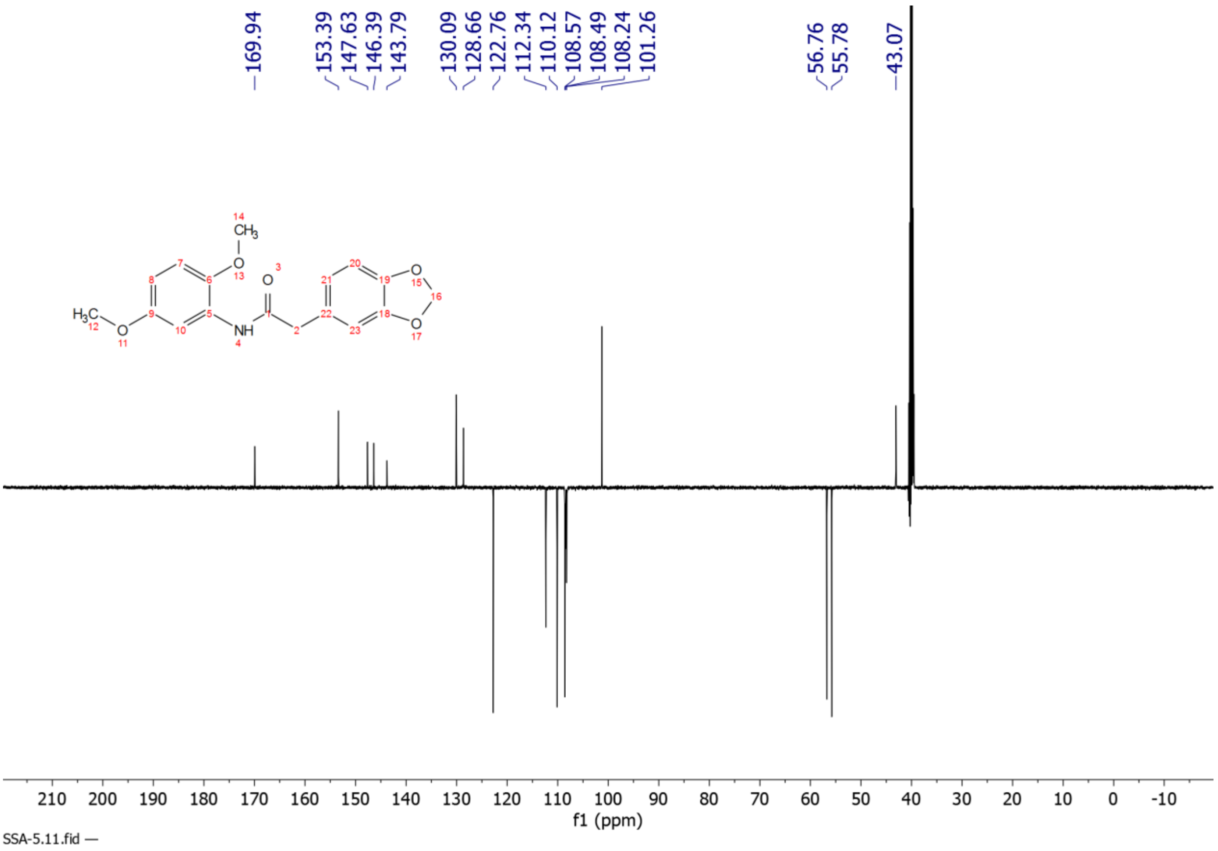

Figure S5. **N-phenylbenzo[d][1,3]dioxole-5-carboxamide (IIa) SSA-10**

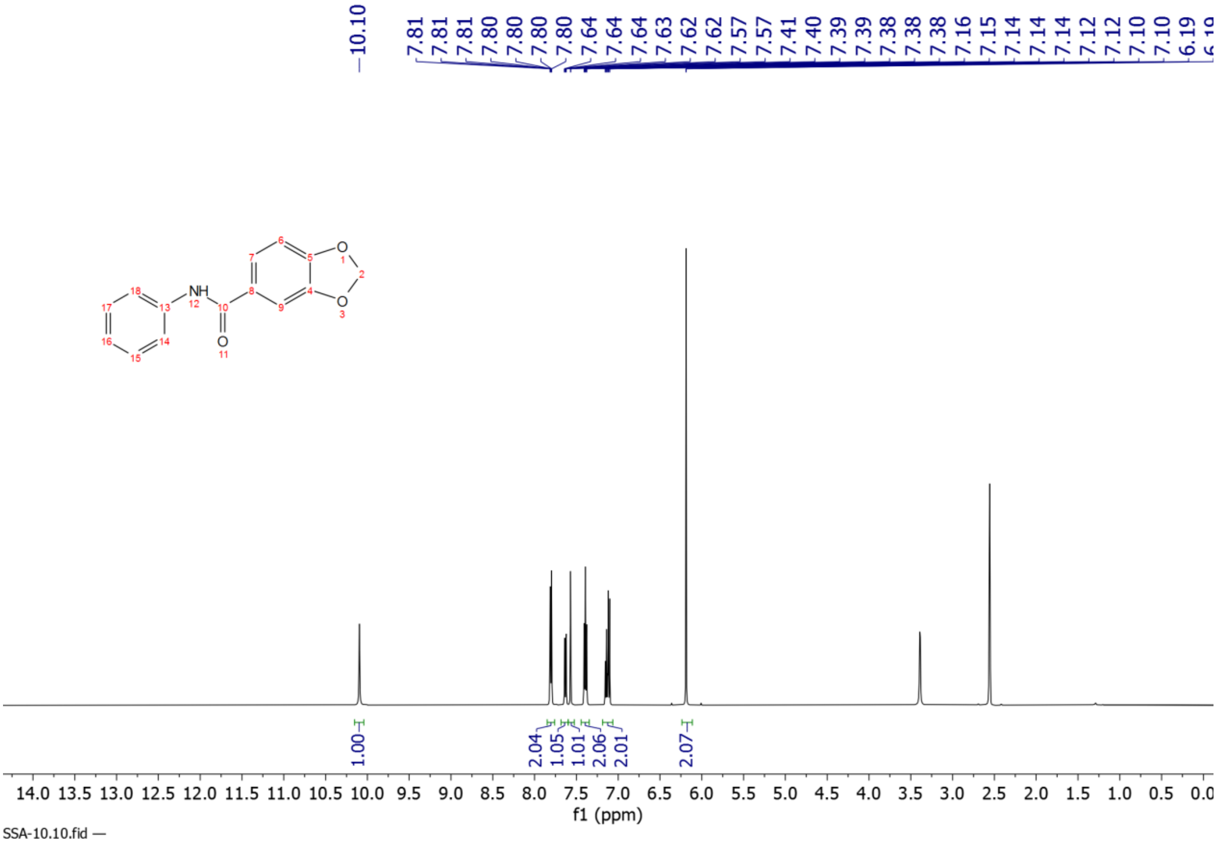

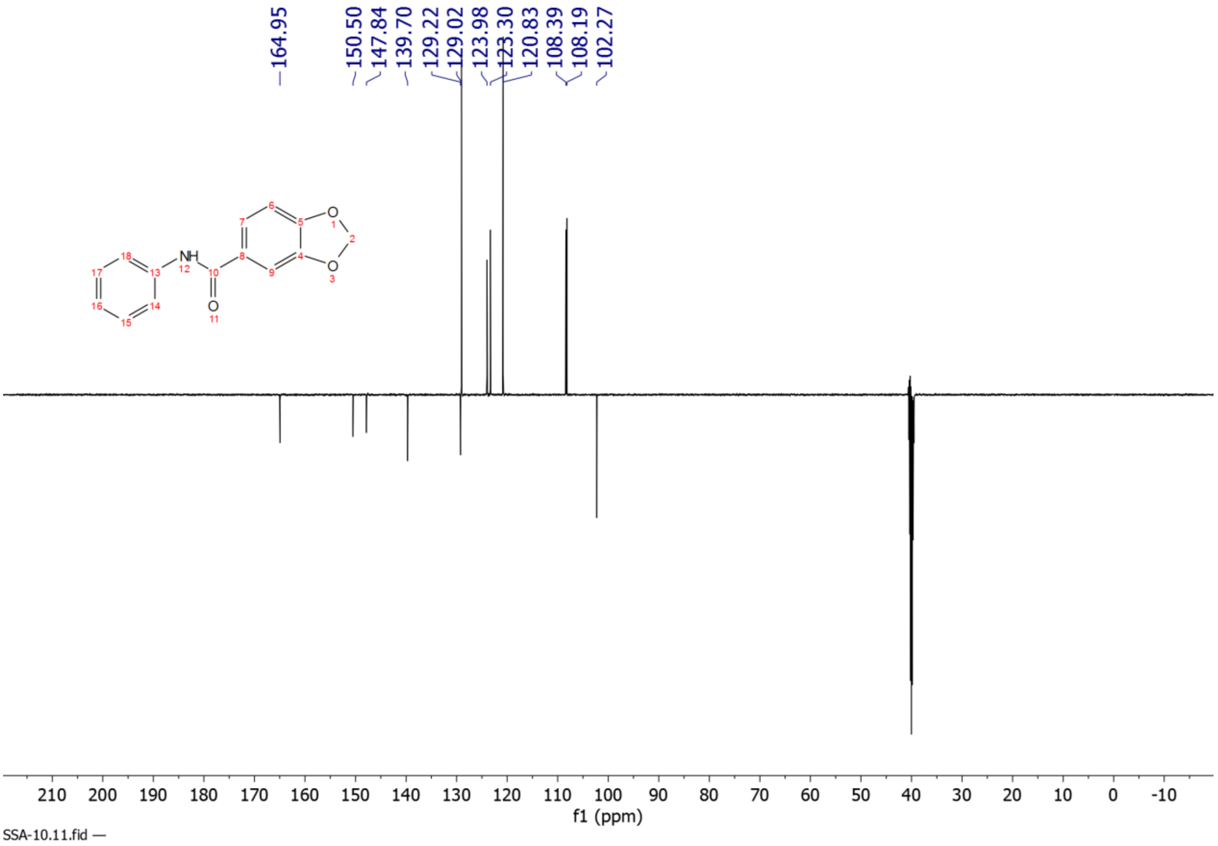

Figure S6. **N-(3-(trifluoromethyl)phenyl)benzo[d][1,3]dioxole-5-carboxamide (IIc)**

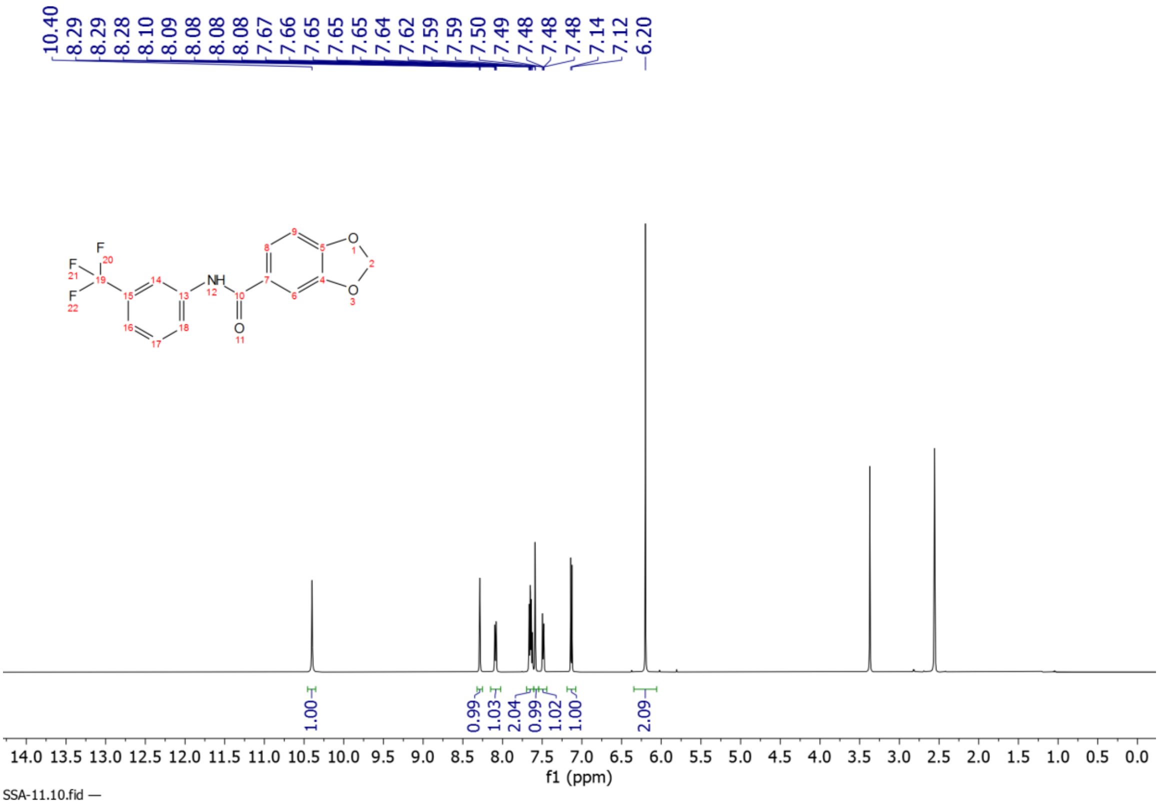

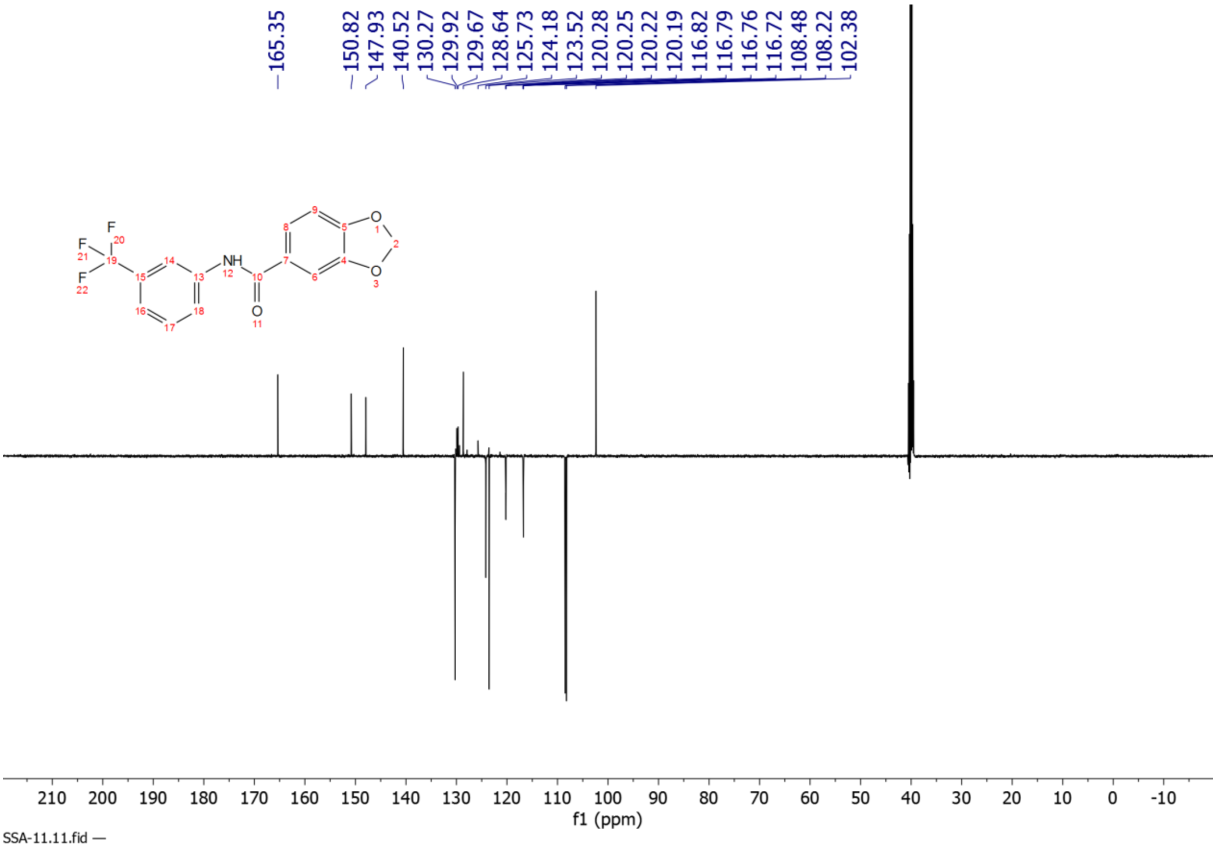

Figure S7. N-(3,4,5-trimethoxyphenyl)benzo[d][1,3]dioxole-5-carboxamide (IIb) SSA-12

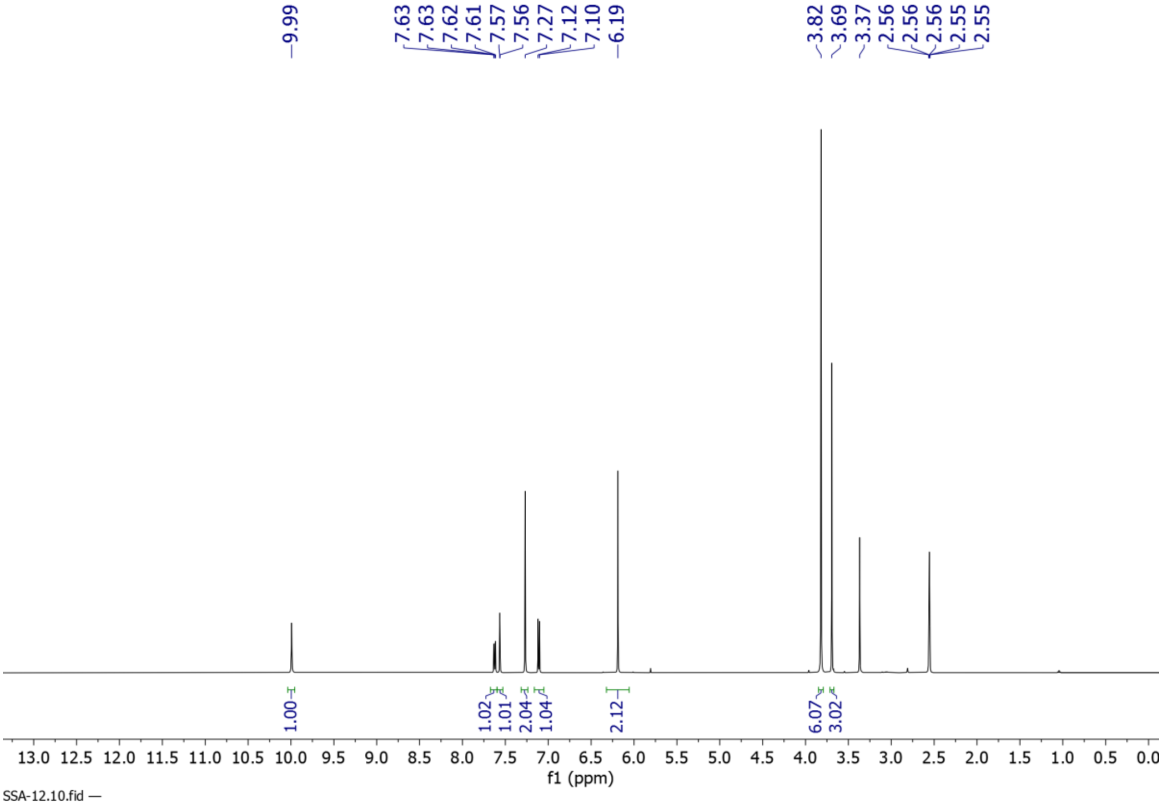

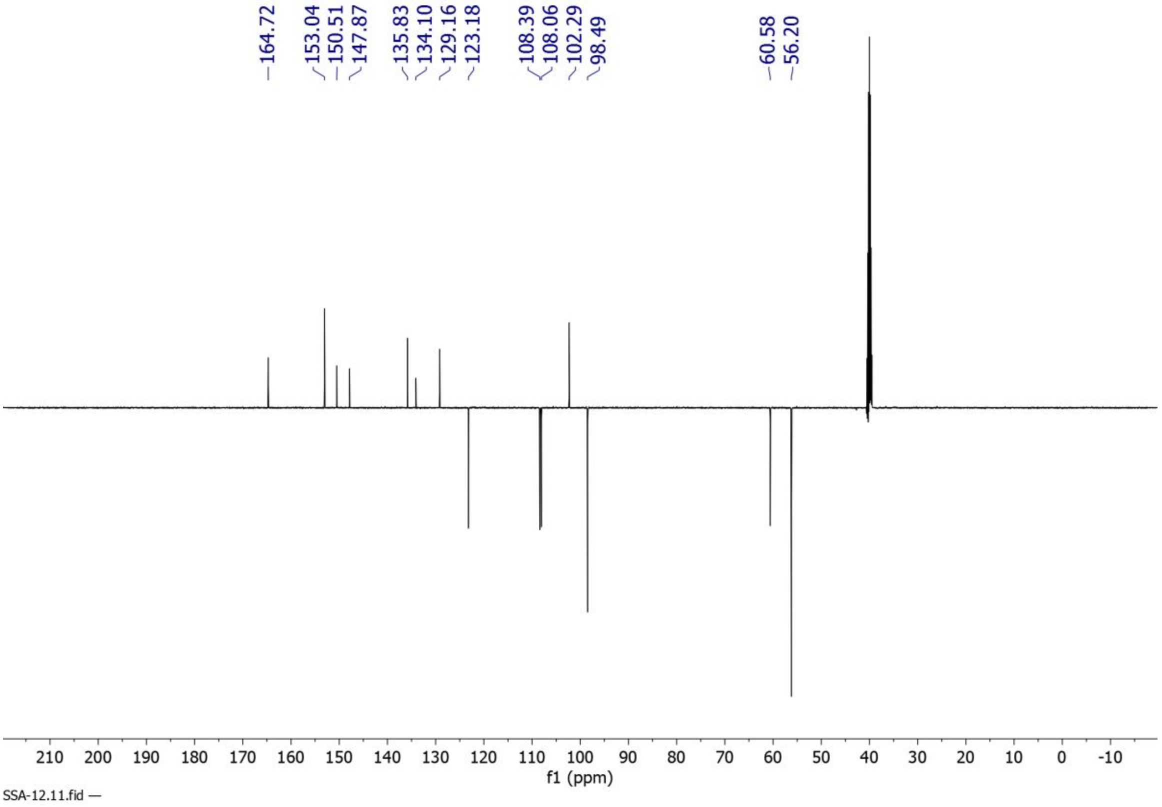

Figure S8. N-(4-(2-methoxyphenoxy)phenyl)benzo[d][1,3]dioxole-5-carboxamide (IId)

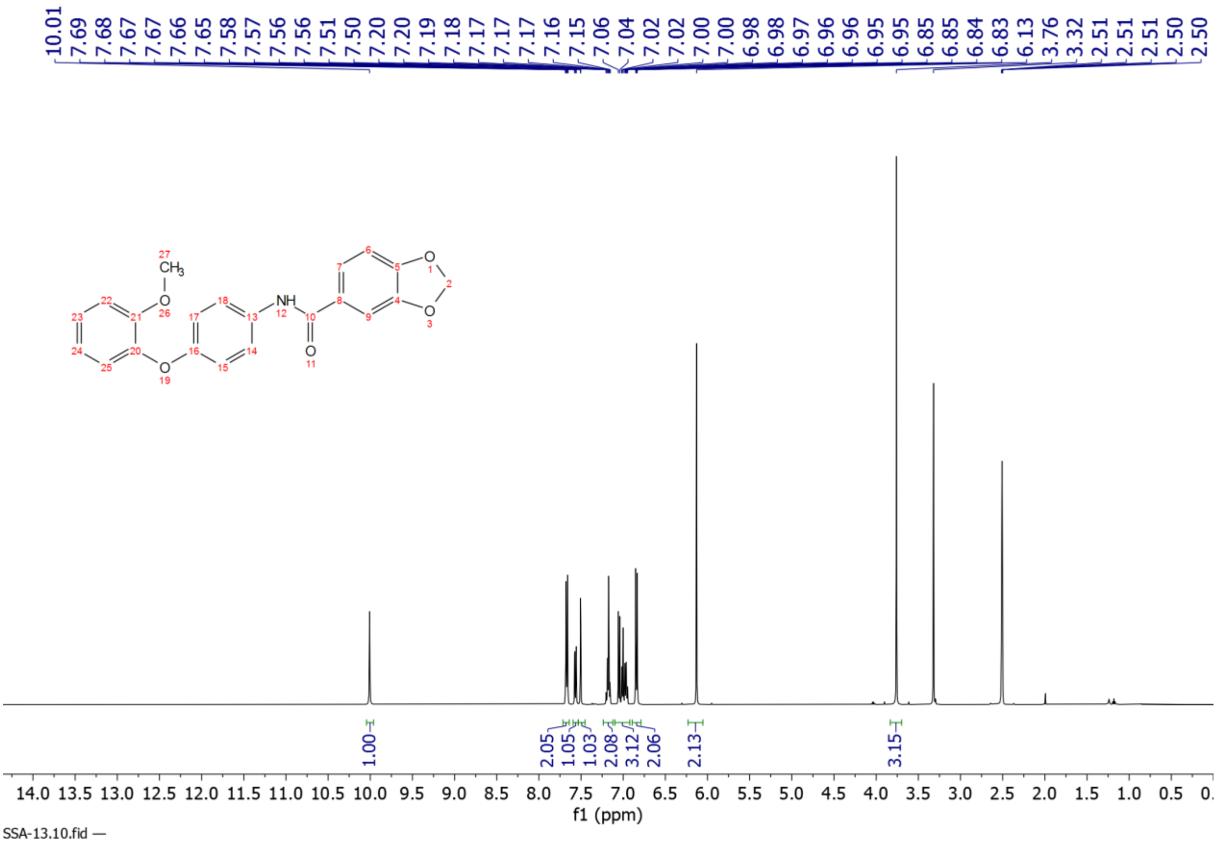

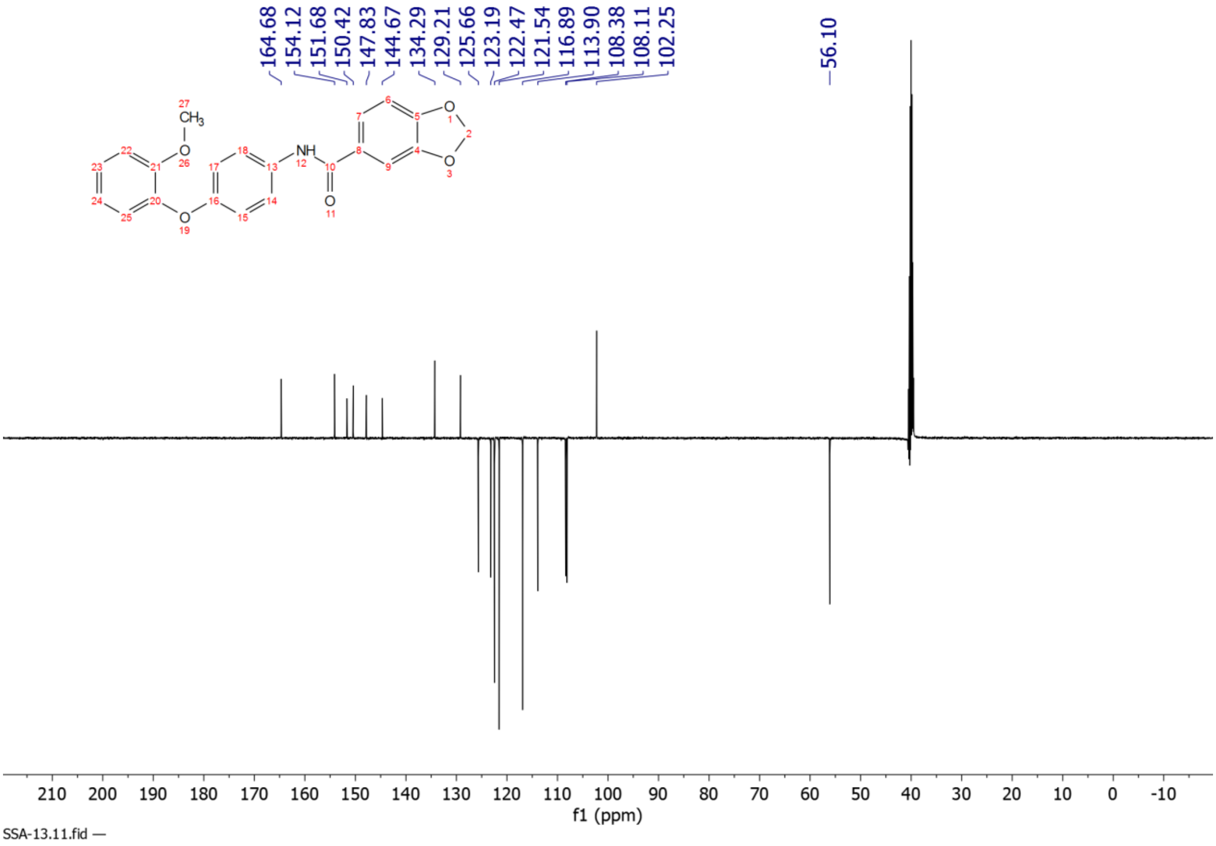

Figure S9. **N-(3,5-dimethoxyphenyl)benzo[d][1,3]dioxole-5-carboxamide (IIe) SSA-15**

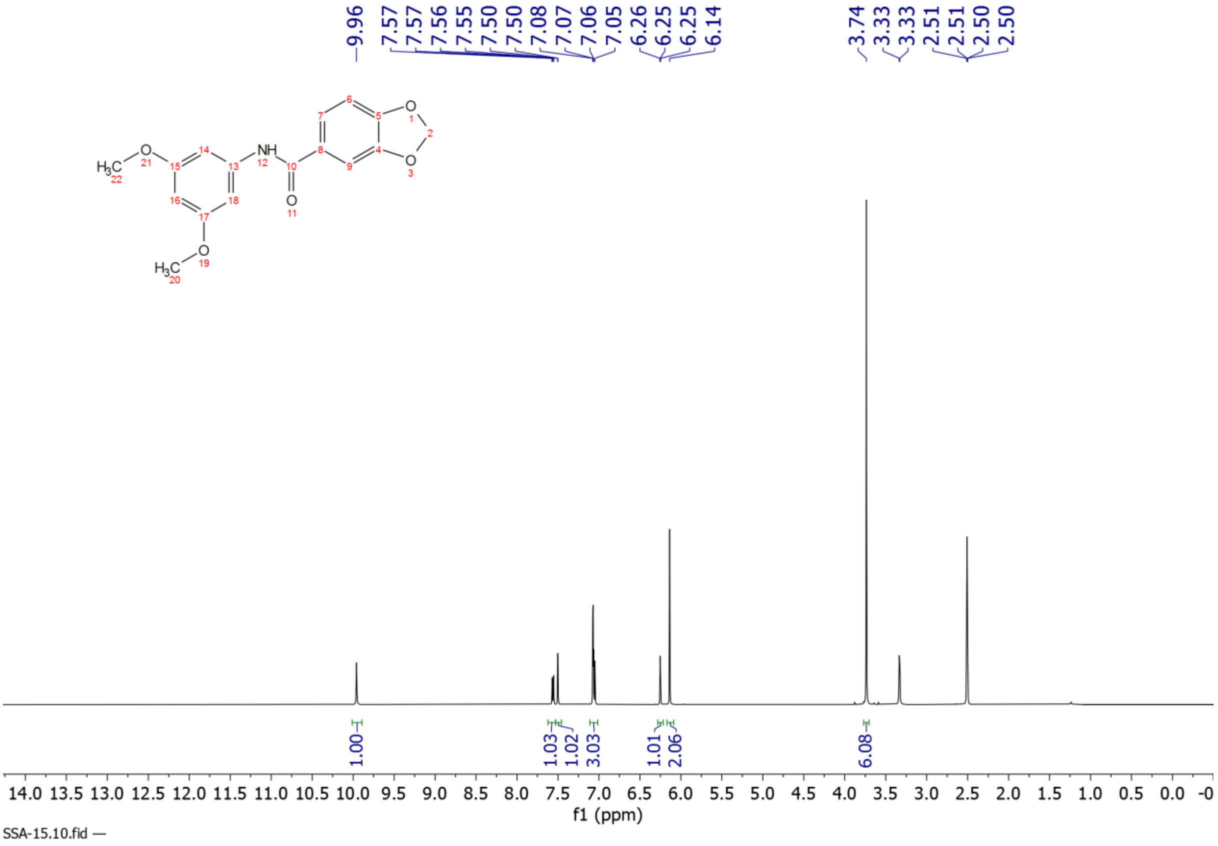

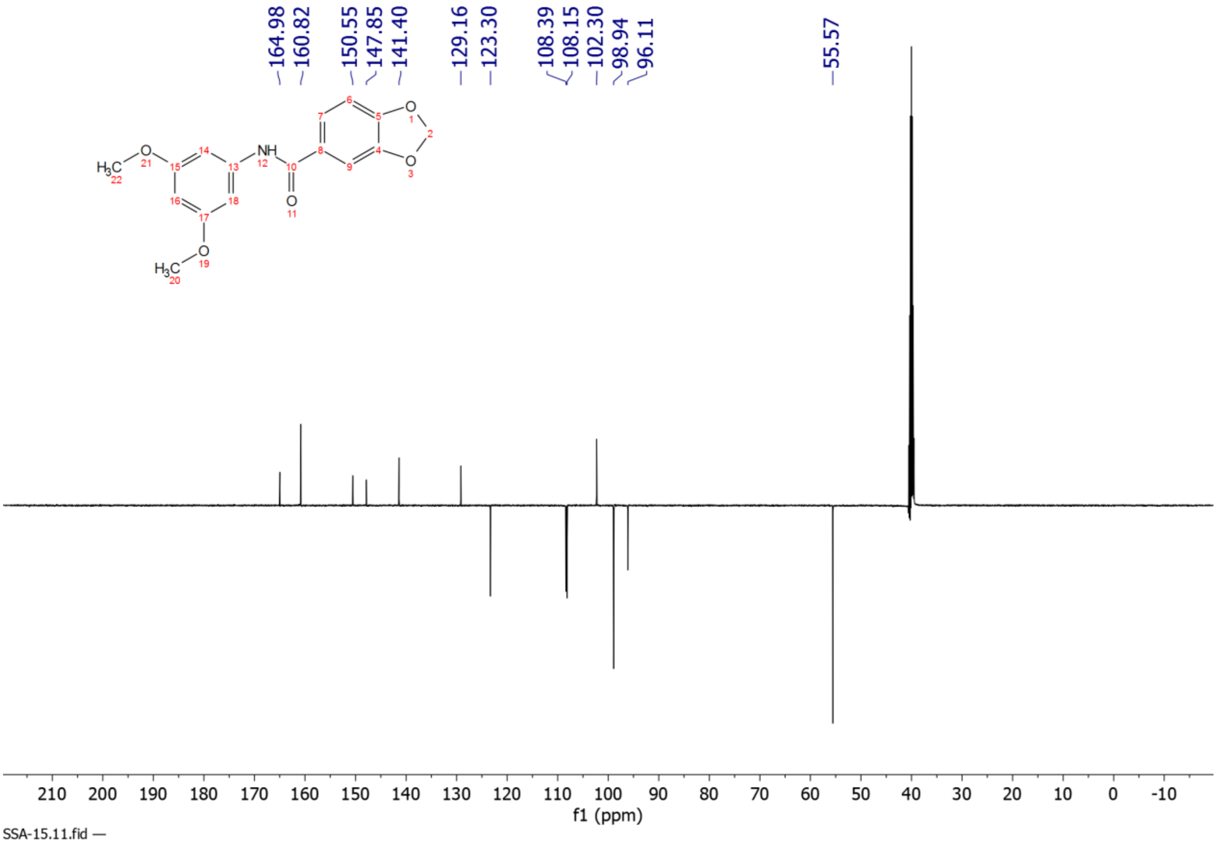

Supplement: Supplementary file 1 [file biomolecules-13-01486-s001.zip › biomolecules-2593454-supplementary.pdf]
